# Comprehensive epigenomic profiling of human alveolar epithelial differentiation identifies key epigenetic states and transcription factor co-regulatory networks for maintenance of distal lung identity

**DOI:** 10.1186/s12864-021-08152-6

**Published:** 2021-12-18

**Authors:** B. Zhou, T. R. Stueve, E. A. Mihalakakos, L. Miao, D. Mullen, Y. Wang, Y. Liu, J. Luo, E. Tran, K. D. Siegmund, S. K. Lynch, A. L. Ryan, I. A. Offringa, Z. Borok, C. N. Marconett

**Affiliations:** 1 Division of Pulmonary, Critical Care and Sleep Medicine, Department of Medicine, Keck School of Medicine, University of Southern California, Los Angeles, CA 90089 USA; 2 Hastings Center for Pulmonary Research, University of Southern California, Los Angeles, CA 90089 USA; 3 Norris Comprehensive Cancer Center, Keck School of Medicine, University of Southern California, Los Angeles, CA 90033 USA; 4 Department of Surgery, Keck School of Medicine, University of Southern California, Los Angeles, CA 90089 USA; 5 Department of Biochemistry and Molecular Medicine, Keck School of Medicine, University of Southern California, Los Angeles, CA 90089 USA; 6 Department of Preventive Medicine, Keck School of Medicine, University of Southern California, Los Angeles, CA 90089 USA; 7 Department of Engineering, Test Manufacturing Group, MAXIM Integrated Products, Sunnyvale, CA 95134 USA; 8 Department of Stem Cell Biology and Regenerative Medicine, Keck School of Medicine, University of Southern California, Los Angeles, CA 90089 USA; 9 Division of Pulmonary, Critical Care and Sleep Medicine, Department of Medicine, University of California, San Diego, La Jolla, CA 92093 USA

## Abstract

**Background:**

Disruption of alveolar epithelial cell (AEC) differentiation is implicated in distal lung diseases such as chronic obstructive pulmonary disease, idiopathic pulmonary fibrosis, and lung adenocarcinoma that impact morbidity and mortality worldwide. Elucidating underlying disease pathogenesis requires a mechanistic molecular understanding of AEC differentiation. Previous studies have focused on changes of individual transcription factors, and to date no study has comprehensively characterized the dynamic, global epigenomic alterations that facilitate this critical differentiation process in humans.

**Results:**

We comprehensively profiled the epigenomic states of human AECs during type 2 to type 1-like cell differentiation, including the methylome and chromatin functional domains, and integrated this with transcriptome-wide RNA expression data. Enhancer regions were drastically altered during AEC differentiation. Transcription factor binding analysis within enhancer regions revealed diverse interactive networks with enrichment for many transcription factors, including NKX2–1 and FOXA family members, as well as transcription factors with less well characterized roles in AEC differentiation, such as members of the MEF2, TEAD, and AP1 families. Additionally, associations among transcription factors changed during differentiation, implicating a complex network of heterotrimeric complex switching in driving differentiation. Integration of AEC enhancer states with the catalog of enhancer elements in the Roadmap Epigenomics Mapping Consortium and Encyclopedia of DNA Elements (ENCODE) revealed that AECs have similar epigenomic structures to other profiled epithelial cell types, including human mammary epithelial cells (HMECs), with NKX2–1 serving as a distinguishing feature of distal lung differentiation.

**Conclusions:**

Enhancer regions are hotspots of epigenomic alteration that regulate AEC differentiation. Furthermore, the differentiation process is regulated by dynamic networks of transcription factors acting in concert, rather than individually. These findings provide a roadmap for understanding the relationship between disruption of the epigenetic state during AEC differentiation and development of lung diseases that may be therapeutically amenable.

**Supplementary Information:**

The online version contains supplementary material available at 10.1186/s12864-021-08152-6.

## Background

Diseases involving the distal lung epithelium, such as chronic obstructive pulmonary disease (COPD), idiopathic pulmonary fibrosis (IPF), and lung adenocarcinoma (LUAD), are major contributors to morbidity and mortality in the United States [1–3] and worldwide. While environmental factors are established contributors to the development and progression of distal lung diseases [4–6], little is understood about how the underlying epigenetic architecture of the adult lung is disrupted in these disease processes. The distal lung epithelium is comprised of two main epithelial cell types, alveolar epithelial type 1 (AT1) and type 2 (AT2) cells, each with distinct physiological roles, morphology, and molecular profiles [7]. Understanding the molecular interrelationship between these two diverse cell types and the distinct role each cell type plays in disease initiation and progression is key to developing approaches to combat diseases of the distal lung.

While differences in gene expression between AT2 and AT1/AT1-like cells cultivated in vitro for several days (AT1-like cells) have previously been profiled [8–12], relatively little is known about changes in the epigenetic state between these two cell types. Previous studies have examined general epigenetic activation and repression states [8], and specific transcription factor interactions with chromatin state [13]; however, there has been no systematic profiling of global epigenomic alterations during AEC differentiation to date. Gene expression can be regulated by either activation or repression. Enhancers are epigenetic regulatory elements that can act at great distances from their target promoters to control activation of gene expression, and can also play a key role in cell type specification and regulation of disease processes [14]. They are characterized by a nucleosome-depleted stretch of DNA that allows for transcription factor binding. This exposed DNA region is flanked by well-positioned nucleosomes decorated with post-translational modifications indicative of active enhancer activity. Specifically, nucleosomes at the site of active enhancers show co-occurrence of histone 3 lysine 27 acetylation (H3K27Ac) and histone 3 lysine 4 mono-methylation (H3K4me1). Open DNA regions within the center of the enhancer region can be interrogated genome-wide using Formaldehyde-Assisted Isolation of Regulatory Elements (FAIRE), followed by massive parallel sequencing [15]. The open region identified by FAIRE is commonly bound by transcription factors that function to regulate downstream target gene expression levels. Often these regions are also found to be depleted of CpG methylation [16].

We set out to discover how epigenomic remodeling of AECs directs the reprogramming of AT2 into AT1 cells during AEC differentiation using a well-characterized 2-dimensional (2D) culture model derived from primary human cells. The 2D model of alveolar epithelial cell differentiation has been in use for over 35 years [17, 18], and has been extensively used by the field [19] to discover and characterize markers of AT2 and AT1 cells [19–25], which have subsequently been confirmed by recent single cell consortia findings using primary cells [11, 26]. The 2D model of AEC differentiation has also paved the way for understanding alveolar responses to injury [27, 28], environmental exposures [29], cellular transport properties [30], and alveolar repair [31]. Strikingly, results initially derived using this model system have been verified in vivo*,* such as the plasticity of AT1 cell differentiation [32–34], which was confirmed later using mouse models [34–37], as well as in the more recently developed 3D organoid model of alveolar formation [34, 38]. It has been over 14 years since this model was translated into human tissue systems [39, 40] and the results generated from studies utilizing this model have shown a high degree of relevance to human lung differentiation and disease [27, 41]. AEC grown in the 2D model are also able to form tight epithelial monolayers consistent with the in vivo lung, which can be measured by transepithelial resistance [18], a property that is not quantifiable in the 3D organoid model. In sum, this model results in AT1-like cells, which recapitulate many of the gene expression patterns, physiological behaviors, and morphological characteristics of AT1 cells found in vivo [42–44]*.*

To characterize epigenomic remodeling during AEC differentiation and its influence on transcriptional patterning, we performed comprehensive profiling of the epigenetic state using histone marks known to affect gene expression and regulation of genomic architecture [45]. We focused our study on enhancers as the epigenetic elements that most influence gene expression during differentiation, and within them we found enrichment for high-confidence transcription factors predicted to bind to these regions and that likely act in concert to direct AEC differentiation. We then utilized the compendium of enhancer signatures across the spectrum of human tissues to identify enhancers and associated transcription factors that were specific for human alveolar epithelial AT2 and AT1-like cells, which can be of future utility in the generation of cell-type specific models of diseases arising from the alveolar epithelium. We present herein a comprehensive profile of epigenetic alterations that occur during AEC differentiation and describe their influence on coordinated gene expression patterning to determine phenotypic transitions between AT2 and AT1-like cells and direct the acquisition of an AT1-like cell fate.

## Results

### Enhancers constitute the major epigenomic alterations during AEC differentiation

**Fig. 1:**
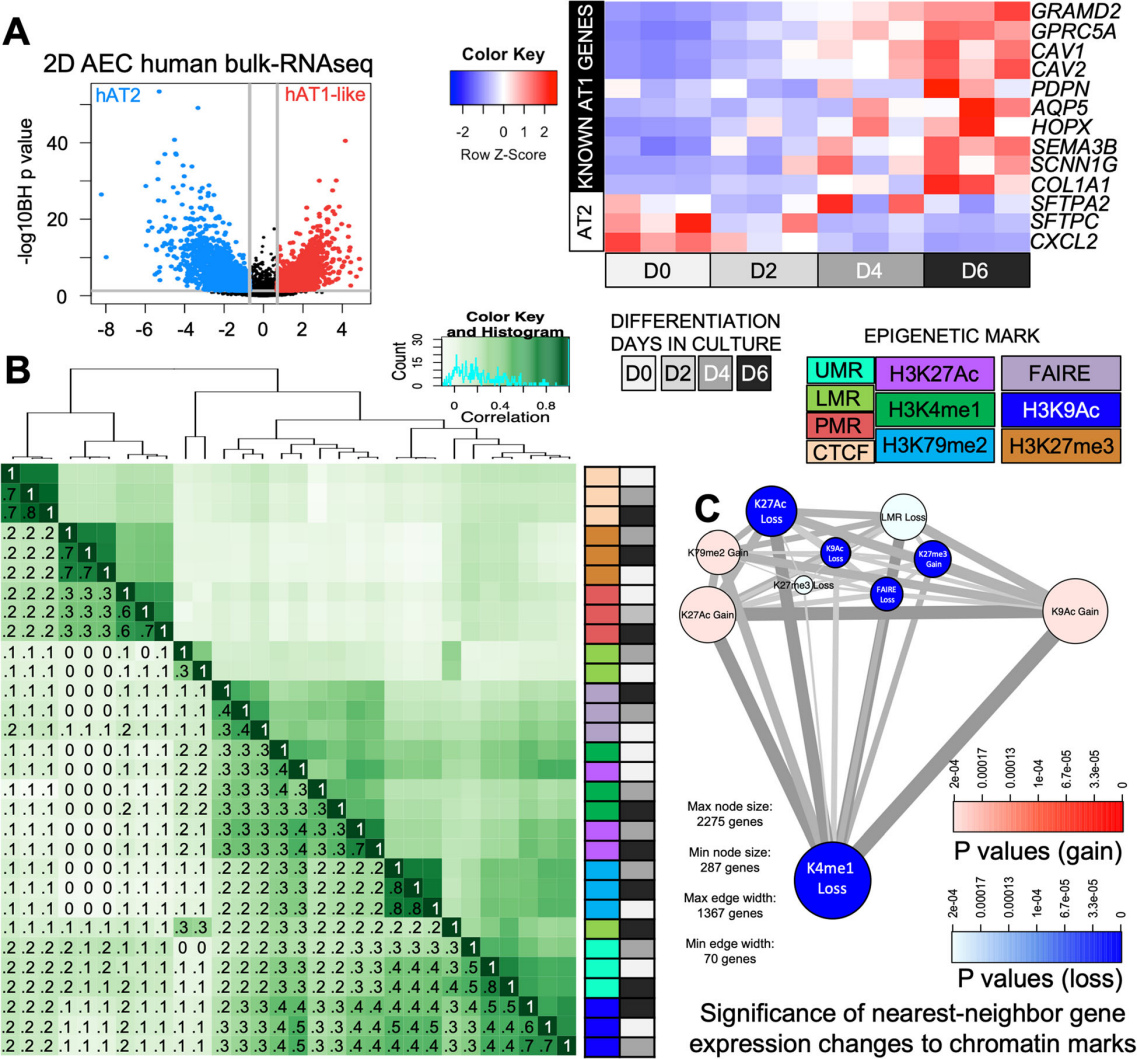
Enhancers constitute the major epigenomic alterations in AEC differentiation. **A**) Left: Volcano plot (left) of differential gene expression during differentiation in the 2D model. Blue = AT2 (D0) enriched genes, red = AT1-like (D6) enriched genes. Right: Heatmap of known known AT1 and AT2 cell marker gene expression in RNAseq from 2D AEC differentiation model at the indicated days. Colors are scaled by row; blue = low expression, red = high expression. **B**) Unsupervised hierarchical cluster analysis of R-squared correlation matrix of chromatin-mark occupancy demonstrates similarity across the major known epigenetic marks. Darker green = more highly correlated genomic distribution, white = less correlated distribution patterns across the genome. Colored annotation panels along the side of the heatmap correspond to the days in culture (greyscale) and epigenetic mark (colors) being measured. UMR = unmethylated regions, LMR = low methylation regions, PMR = partially methylated regions. Numbers on heatmap indicate R correlation value rounded to nearest tenth. **C**) PIANO diagram showing correlation between loss or gain of epigenetic mark and changes in expression of the nearest-neighbor gene. Red scale = significance of enrichment for genes with gain in expression during AEC differentiation, blue = significance of enrichment for genes with loss in expression during AEC differentiation. Increasing size of circle containing epigenetic mark = more regions associated with gene expression changes, smaller size of circle = fewer epigenetic regions associated with gene expression changes. The thickness of the grey connecting lines indicates the number of genes that are associated with both epigenetic marks, thicker = more genes associated with both marks, thinner = fewer genes.

To determine the relationship between epigenetic alterations and AEC differentiation, we first undertook comprehensive epigenomic profiling of human AEC during differentiation from AT2 to AT1-like cells. AT2 cells were extracted from explant donor lungs that had no prior evidence of chronic lung disease and allowed to differentiate into AT1-like cells in vitro over the course of 6 days utilizing well-established protocols [8, 46]. Preestablished quality control measures ensured that the AEC differentiated appropriately in 2D culture (**Fig. S****1**). Next, the AT2 cell population (D0), transitional AEC (D4), and AT1-like cells (D6) underwent DNA isolation for whole genome bisulfite sequencing (WGBS) (1 million cells each), chromatin fixation for ChIP-seq (5 million cells each ChIP) and corresponding RNA isolation for bulk RNA-seq (1 million cells each) to correlate altered epigenetic states with changes in gene expression from the same population of cells. RNA-seq from the 2D AEC differentiation model underwent differential expression analysis (Fig. 1A) which demonstrated that known AT1 and AT2 cell markers were enriched in the D6 AT1-like cell population. Additionally, genes differentially expressed in the 2D AEC differentiation model were compared to recently published single cell RNA-seq datasets generated by three separate consortia (**Fig. S****2**). Concordance between the 2D AEC differentiation model and AT1/AT2 cell markers within primary human and mouse lung single cell analysis was highly significant (*p* < 2.2^e-16^ for all three consortia findings), and 62 genes were identified as AT1 cell enriched across four datasets (**Supplemental Table** **1**), including known AT1 cell genes *AGER*, *CAV1*/*CAV2*, *CLDN18*, *CLIC3*/*CLIC5*, *GPRC5A*, *HOPX*, *IGFBP7*, *PDPN*, *RTKN2*, *SEMA3B*/*SEMA3E*, and *SPOCK2* [7, 10, 34, 42, 47].

For ChIPseq, antibodies were directed against histone modifications associated with euchromatin (H3K4me1, H3K27Ac, K3K9Ac) and facultative heterochromatin (K3K79me2/3, H3K27me3) marks, as well as the three-dimensional chromatin organizing protein, CCCTC-binding factor (CTCF). During the ChIP process, non-protein bound DNA fragments in the supernatant were collected as “free DNA” and profiled using FAIRE-seq to determine open genomic regions. Inspection of the ratio of peak enrichment to input background revealed that the ChIP-seq data were of acceptable quality for subsequent data analysis (**Figs. S****3****-S****5**). We also determined whether maximal peak occupancy was reached by subdividing ChIP-seq datasets and re-performing peak calling analysis to generate a curve for determining maximal peak occupancy (**Figs. S****3****-S****5**). Our samples had reached the plateau for the number of peaks called, indicating that our sequencing depths were sufficient and had captured the vast majority of the binding sites for the given antibodies. Of note, data quality as measured by peak enrichment from Donor 1 was slightly better than Donor 2, and was therefore used as the discovery dataset, with Donor 2 used as the validation set. The genomic distribution of each epigenetic signature was then mapped back to the hg19 genome and the correlation between samples and the distribution of each mark was determined (Fig. 1B).

WGBS data underwent DNA methylation domain-calling using MethylSeekR [16], which segregates the genome into specific domains based on their level of methylation. Unmethylated regions (UMRs) have less than 10% methylation levels, extend over regions > 10 kb, and have been associated with loci important for cell fate determination [48–50]. Low-methylated regions (LMRs) have between 10 and 30% methylation levels and are associated with active enhancers [51]. Partially methylated domains (PMDs) have between 30 and 70% overall methylation levels, tend to stretch for many kilobases (kb), and are associated with polycomb complex and facultative heterochromatin [52]. The last category which is not explicitly defined by MethylSeekR comprises fully methylated domains (> 70% methylated) which are associated with constitutive heterochromatin. We integrated our WGBS domain data with the ChIP-seq data using the Diffbind package in R to calculate and visualize a correlation matrix of peak overlaps and found that partially methylated regions (PMRs) in AECs were more closely associated with the repressive chromatin mark H3K27me3 and the insulator CTCF (Fig. 1B). CTCF acts as a long-range homodimeric insulator that regulates three-dimensional chromatin structure [53, 54]. Each mark within this group clustered with itself rather than clustering together by differentiation day, indicating that these marks did not undergo major shifts during AEC differentiation.

The remaining histone chromatin marks clustered separately as active chromatin regions. UMRs clustered with the H3K9Ac mark of generalized euchromatin activation. H3K79me2, which is a mark of transcriptional elongation, is segregated as its own smaller cluster within the active enhancer cluster as well. The LMR regions of D6 clustered within the active histone marks, but D0 and D4 LMR regions did not cluster with either repressive or active histone marks. The remaining cluster observed consisted of H3K4me1, H3K27Ac, and FAIRE signal, all marks associated with active enhancers. Interestingly, these H3K4me1 and H3K27Ac marks clustered by AEC differentiation state (i.e., days in culture) instead of by epigenetic mark, indicating that, *genome-wide*, there were substantial changes in the distribution of active enhancers as AT2 cells transition toward an AT1-like cell fate.

We previously observed that the process of AT2 to AT1-like cell differentiation alters the expression of thousands of genes [8]. To further interrogate the genome-wide relationship between epigenetic state and gene expression during in vitro AEC differentiation, we utilized the PIANO package, which performs comparative gene-set enrichment analysis between custom datasets [55]. We compared the gain or loss of each epigenetic mark profiled against changes in the HOMER-annotated nearest neighbor gene expression as a rough measure of association, with the caveat that enhancers can often target genes across great distances and in addition the rate of nearest-neighbor enhancer interaction varies across tissues and development [56] (Fig. 1C). We observed that loss of H3K4me1, H3K27Ac, H3K9Ac, FAIRE, and gain of H3K27me3 were all highly significantly correlated to loss of nearby gene expression from differential RNA-seq analysis during differentiation (blue, all had *p* < 3.3 × 10^− 5^). Loss of LMR signal was also significantly correlated to loss of gene expression, albeit to a lesser extent than the other marks (*p* < 2.0 × 10^− 4^). The gain of H3K27Ac, H3K9Ac, and H3K79me3 were significantly correlated with increases in expression of nearby genes (red). None of the other epigenetic marks were significantly associated with changes in nearby gene expression. We therefore focused on enhancer and open FAIRE regions that were associated with gene expression alterations as a means of identifying key transcriptional regulators during AEC differentiation.

### Identification of FOX family, STAT family, TEAD family, and AP1 complex members as transcription factors changing during AEC differentiation in FAIRE-occupied regions

**Fig. 2:**
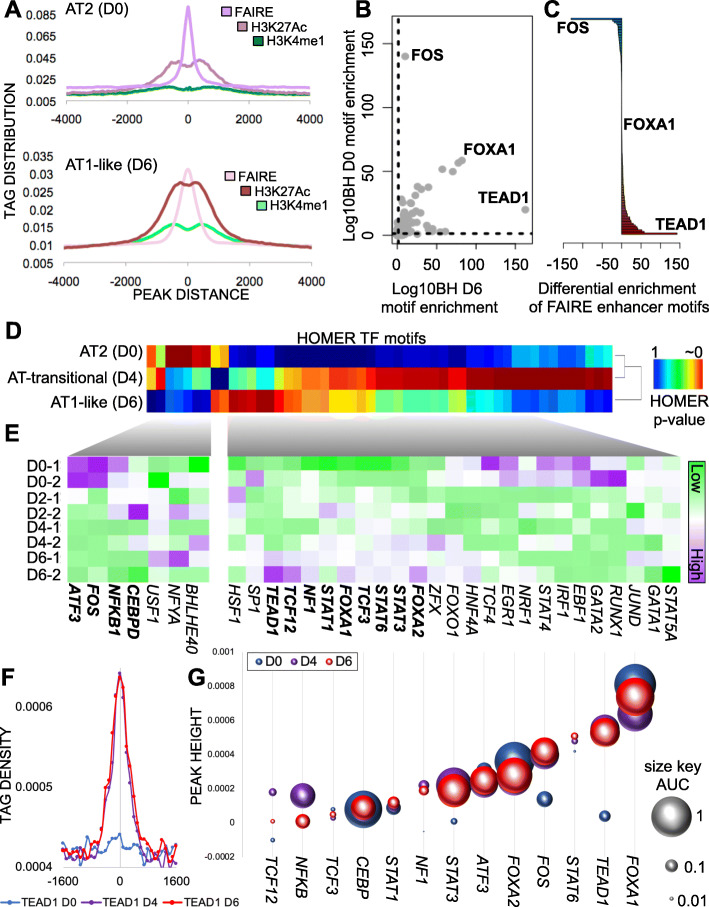
Identification of FOX family, STAT family, TEAD family, and AP1 complex members as transcription factors changing during AEC differentiation in FAIRE-occupied regions. **A**) Enrichment of tag density from center of the epigenetic mark for both AT2 (top) and AT1-like (bottom) cells. **B**) HOMER-computer enrichment of TFBS in AT1-like (X-axis, D6) or AT2 (y-axis, D0) cells. Dotted line indicates -log10BH cutoff for significance. **C**) Distribution of all TFBS predicted motifs available in HOMER and their enrichment in AT1-like (red) vs. AT2 (blue) cells. The BH-corrected *p* value for each TFBS motif was computed in each cell type and AT2 cell enrichment was subtracted from AT1-like cell enrichment. TFBS motifs were then arranged from most AT2-cell specific (top) to most AT1-like cell-specific (bottom). **D**) Unsupervised hierarchical clustering of TFBS enrichment in FAIRE-occupied regions. Red = highly significantly enriched for the indicated TFBS, blue = not significantly enriched. Rows are scaled based on *p* values of motif enrichment significance. **E**) Supervised clustering analysis of gene expression changes for the indicated transcription factors during AEC differentiation. Purple = high expression, green = low expression, each column color scaled by standard deviation within the row. Transcription factors bolded have loss of predicted TFBS and loss of gene expression (AT2 cells, top cluster), or gain of predicted TFBS and corresponding increase in gene expression (AT1-like cells, bottom cluster). **F**) Tag density of TEAD1 motif enrichment within AT2 (blue) or AT1-like (red) FAIRE peaks. Tag densities are centered on the middle of the FAIRE peak (position 0) and normalized by millions-mapped. **G**) The peak height (Y axis) and area under the peak (size of circle) were calculated for all AT2 (blue) and AT1-like (red) enriched TF motifs in FAIRE peaks. TFs were ranked based on AT1-like peak height (smallest to largest)

To determine the quality of enhancer-bound chromatin mark enrichment, we plotted the overall tag density of the enhancer-associated marks FAIRE, H3K27Ac, and H3K4me1 centered on the distance from the middle of the calculated peak region (Fig. 2A). We saw a significant enrichment of FAIRE signal at the center of each predicted enhancer, indicating that open regions were centered around transcription factor footprints as previously reported [57–59]. In addition, we saw a bimodal distribution of H3K4me1 and H2K27Ac spaced ~+/− 100 bp from the center of the peak, indicating nucleosomal positioning consistent with known enhancer elements as well as enrichment of enhancer-associated marks. The enrichment signal faded at ~+/− 2000 bp from the center of the peak, indicating that, on average, epigenetic signals for enhancer regions extended no further than ~ 4 kb. As FAIRE data most closely capture TF binding footprints in between the enhancer-decorated nucleosomes, we utilized the FAIRE data in both AT2 (D0) and AT1-like (D6) cells to examine the relative enrichment for all predicted TF motifs contained in the HOMER database (Fig. 2B). We observed that the motifs for the TF FOS and, to a lesser extent, similar members of the AP-1 family, were the most statistically significant in the AT2 cell FAIRE regions. In contrast, we identified several TF motifs that were highly significantly enriched in AT1-like FAIRE samples, most prominently TEA domain family member - 1 (*TEAD1*). Notably, there were several TF motifs enriched in both cell types, such as forkhead box protein A1 (*FOXA1*), indicating that FOXA1 may exert its function as a pioneering TF in both cell types. To identify those motifs which demonstrated cell-type preference, we performed subtractive analysis between AT1-like and AT2 cell motif enrichment (Fig. 2C). This demonstrated that the TEAD motifs were much more significantly enriched in the AT1-like cell motifs consistent with recent reports [13]. FOS was the most significantly enriched motif in the FAIRE open regions of AT2 cells. We previously observed FOS motif enrichment in genomic regions that were open and decorated with H3K9Ac in AT2 cells that then became closed and covered in the H3K27me3 repressive mark in AT1-like cells during AEC differentiation [8].

Once we had determined the statistical enrichment of TF motifs within the FAIRE-identified open regions for each cell type, we correlated those motifs to the expression levels of their corresponding gene. Only a handful of motifs were significantly enriched in AT2 cells and not in AT1-like cells (Fig. 2D). Comparison of these predicted altered binding sites with gene expression changes throughout differentiation yielded subsets of TFs where motif enrichment in enhancers decreased along with loss of TF expression (Fig. 2E). This set of TFs included CCAAT-enhancer binding protein delta (*CEBPD*), nuclear factor kappa-B (*NFKB*), FOS and activating transcription factor-3 (*ATF3*). Consistent with these results, our previous work demonstrated a decrease of NFKB and FOS signaling during AEC differentiation [8]. In contrast, we observed increased RNA expression during AEC differentiation and increased motif enrichment for *FOXA1*, *FOXA2*, signal transducer and activator of transcription (*STAT1*/*STAT3*/ *STAT6*), nuclear factor 1 (*NF1*), transcription factor 3/12 (*TCF3/TCF12*), and *TEAD1* (Fig. 2E). Previous work in our laboratory and others has demonstrated a role for FOXA1/2 and Wnt signaling in AEC differentiation [8, 60, 61]. Recently published work has also identified a key role for TEAD as the downstream target of YAP/TAZ signaling in establishment and maintenance of AT1 cell phenotypes [13].

To further refine and rank candidate TFs involved in AEC differentiation we calculated the peak height and area under the peak for each predicted TF motif in the FAIRE regions in AT2 (D0), AT-transitional (D4) and AT1-like (D6) cells (see example for *TEAD1* in Fig. 2F). TFs with strong signals near the center of a FAIRE peak [62], that can displace histones and create the FAIRE open region signal, would be detected using this method,. However, we also observed this method working for non-pioneering TF, such as *TEAD1* on D4 (purple) and D6 (red). Conversely, lack of a discernable peak near the center of the FAIRE region would argue against a functional relationship between the FAIRE open region signal and TF binding, as we observed for *TEAD1* in AT2 cells (blue). We then ranked all TFs from smallest footprint to largest footprint as a measure of predictive strength of involvement (Fig. 2G). We observed that the HOMER calculated *p* value did not perfectly correlate to peak enrichment at the center of the FAIRE peak, arguing that using only the p value calculations to assign involvement of a TF may over-interpret the involvement of a given TF in the pathway being studied. Using enrichment at the center of the FAIRE peak as a metric for ranking TFs, we observed the pioneering TF *FOXA1* as the top-enriched candidate in FAIRE-marked open regions in D0, D4, and D6 cells. Overall, D4 and D6 motif enrichment for these top factors was highly correlated, with the notable exceptions of *NFkB* and *TCF12*. Additionally, TEAD showed the largest change between D0 and D4/D6. In sum, we identified several TFs that are predicted to regulate enhancer dynamics and cellular phenotype during AEC differentiation. These results suggest that rather than a single factor, a network of TFs is coordinated in a temporal fashion to orchestrate AEC reprogramming and gene expression changes requisite for AT1-like cell fate.

### Transcription factor interaction networks within enhancer regions shift during AEC differentiation

**Fig. 3:**
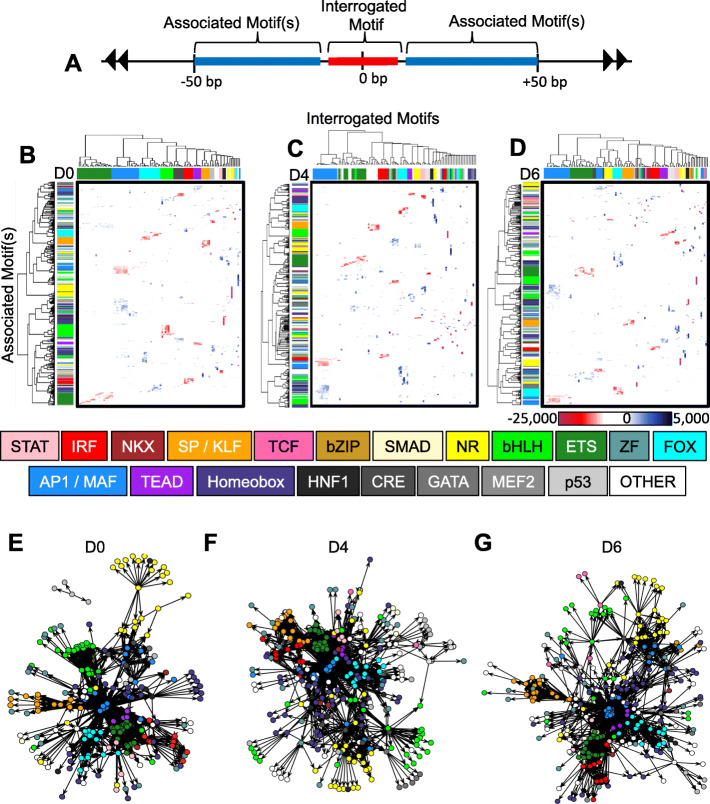
Transcription factor interaction networks within enhancer regions shift during AEC differentiation. **A**) Diagram of AEC enhancer regions selected for further study. Regions centered around the top 100 significantly enriched motifs within each cell type, dubbed “Interrogated Motifs”, colored red. 50 bp regions adjacent to the Interrogated Motif were subset (blue regions) to identify “Associated Motifs” that were significantly associated with the Interrogated Motifs. **B**-**D**) Unsupervised hierarchical clustering in AT2 cells (**B**) D4 AT-transitional cells (**C**), and AT1-like cells (**D**) of top 100 Interrogated Motifs (columns) and predicted Associated Motif significant interactions (rows). All HOMER TFBS were included in the analysis. Within the heatmaps, red indicates binding sequence similarity to the Interrogated Motif (positive control), blue indicates Associated Motifs had distinct core binding sequences, white indicates motif enrichment was not statistically significant. Families of TFs with similar core binding sequences were labeled with a distinct color to visually discern motif association patterns (column and row colors labels). **E**-**G**) Network analysis of AT2 (**E**), AT-transitional (**F**) and AT1-like (**G**) enhancer TF interactions. Each circle represents a “node”, or specific TF. Families of TFs are similarly colored according to the central key within the figure. Significant association is denoted by connecting ‘edges’, ie., lines (AT2: p < e-50; AT1-like: p < e-100). Length of edge/line is not indicative of significance level, all associations above the indicated thresholds are shown

To confirm our observations that a large number of TFs were significantly enriched in AEC enhancer regions and associated with distinct sets of differentially expressed genes during differentiation, we applied knowledge from the biochemistry field about the spacing between heterodimeric TF complexes that bind site-specifically to DNA to understand how TF families were changing their associations during AEC differentiation. The majority of characterized TF heterodimeric interactions are thought to occur between binding partners that rest on DNA within 50 bp of each other, based on many decades of steric and mutational analyses [63–65]. Therefore, we began by running HOMER transcription factor binding site (TFBS) prediction on AT2 and AT1-like enhancer regions. Next, we annotated where all of the top 100 significantly enriched motifs in each cell type sat within their respective enhancers. In many cases, multiple instances of a given motif were found in a given enhancer region. To reduce overrepresentation of these regions, we set a cut-off of up to 10 motif instances in a given enhancer, which encompassed over 99% of all significantly enriched TF motifs from our initial list of top 100, hereafter referred to as the “Interrogated Motif” (Fig. 3A). Next, we ran HOMER on the 100 bp region surrounding the Interrogated Motif to determine which TF families occurred as “Associated Motifs” within that 100 bp window (blue regions, Fig. 3A). Inclusion of the Interrogated Motif allowed for a positive control (red region, Fig. 3A). Next, results from all 100 Interrogated motifs in AT2 cell enhancer regions (D0, Fig. 3B), AT-transitional (A4, Fig. 3C) and AT1-like cell enhancer regions (D6, Fig. 3D) underwent unsupervised hierarchical clustering.

The resultant heatmaps, showing the Interrogated Motifs as columns and Associated (secondary) Motifs as rows, showed several TF motif associations, but overall there was a large divergence in Associated Motif associations over days in culture. As expected, family members with a similar core motif sequence displayed similar enrichments for Associated Motifs, i.e., all ETS family members with the core CAGGAA sequence were predicted to have similar Associated Motif partners. This resulted in clusters of blue colored-Associated Motif families that were associated with the primary motif. Interestingly, AP1 and MAF family members share the same core TGAxxTCA sequence but differ widely in their Associated Motif association (Fig. 3B). AP1 family members were tightly associated with ETS, FOX, and members of the basic Helix-Loop-Helix family, whereas significant association of nuclear receptors (NRs) as Associated Motifs was only observed in MAF family member Interrogated Motifs. ETS family members, and in particular Evt5, have been shown to regulate AT2 cell fate as well as FOX factors, validating these findings [66, 67]. Also, in AT2 cells, TEAD family member Associated Motifs with a core sequence GGAAT were found nearby AP1 family Interrogated motifs. This is in contrast to FAIRE results, which showed enrichment for TEAD family members within FAIRE regions only within AT1-like cells.

Strikingly, AT-transitional (D4) cells showed more diffuse clustering of motifs (Fig. 3C), indicating that dynamic motif shifting may be occurring in a temporally controlled manner. AT1-like D6 cells also showed many more connections between Interrogated Motif and Associated Motif families than AT2 cells. Some families, such as TCF and SMAD that were not detected in AT2 cells, were now significantly associated with multiple Interrogated Motifs. Beyond this, many families of TFs split, so that different family members, with a nearly identical core binding sequence, showed drastically altered associations with Interrogated Motifs on D4. Motif families began to segregate by D6 but did not fully stabilize to the level observed in AT2 (D0) cells.

The high degree of interconnectivity between TF Interrogated and Associated Motifs led us to utilize a network clustering framework to visualize the degree of interaction among these families of transcription factors and how these relationships changed during differentiation. Network analysis [68, 69] was performed on the TFBS Interrogated and Associated Motifs in AT2 (D0) cells (significance cut-off: p < e^− 50^, Fig. 3E), AT-transitional (D4) cells (significance cut-off: *p* < 10^− 100^, Fig. 3F) and AT1-like cells (significance cut-off: p < 10^− 100^, Fig. 3G). Results indicated that AP1 family members formed the centralized node of for D0 and D6, but that D4 also contained NKX, TEAD, FOX, and bHLH factors at the center of the network. In sum, we observed that many transcription factor families, representing dozens of individual TFs, changed their predicted interactions during 2D AEC differentiation.

### Associated motif interaction networks for AP1/MAF, TEAD, and FOX families in enhancer regions

**Fig. 4:**
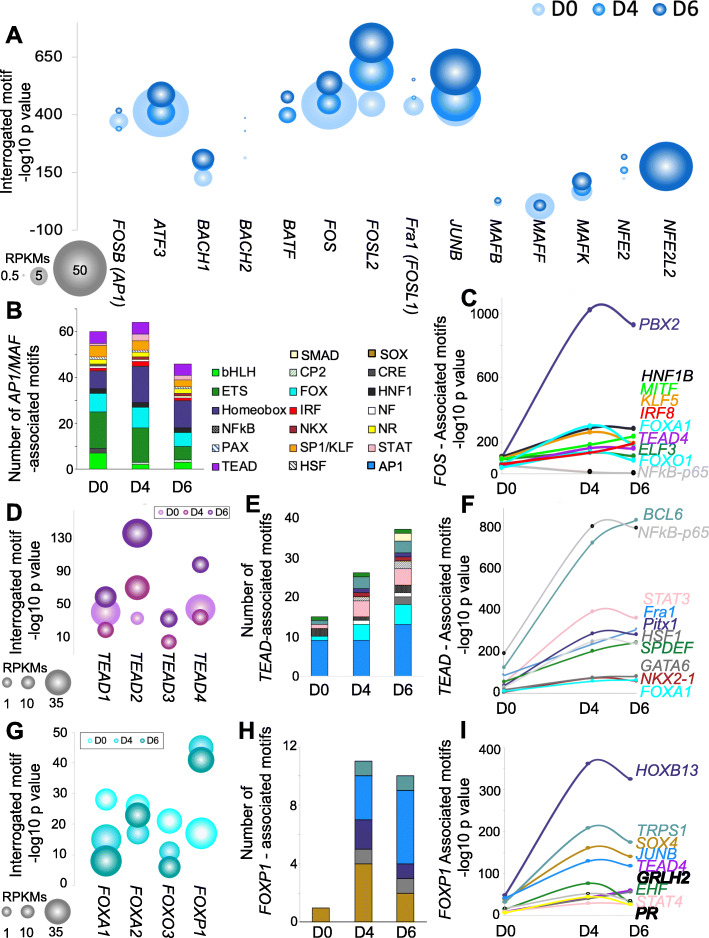
Associated motif interaction networks for AP1/MAF, TEAD, and FOX families in enhancer regions. **A**) Dot plot indicating significance of the indicated interrogated motif in the AP1/MAF family of TFs at either D0 (light blue), D4 (medium blue), or D6 (dark blue). Size of dot is reflective of RPKM expression level at the indicated day. RPKMs are an average of all three donors profiled by RNAseq. **B**) Stacked histogram of factors whose motifs were associated with the AP1/MAF family member *FOS*. TF families are colored according to conserved binding sequence. **C**) Significance of the indicated TFBS motif associated with FOS in D0, D4, and D6 enhancer regions. A representative factor from each family of TFs is shown. **D**) Dot plot indicating significance of the indicated interrogated motif in the *TEAD* family of TFs at either D0 (lilac), D4 (purple), or D6 (indigo). Size of dot is reflective of RPKM expression level at the indicated day. RPKMs are an average of all three donors profiled by RNAseq. **E**) Stacked histogram of factors whose motifs were associated with the TEAD family member *FOS*. TF families are colored according to conserved binding sequence as in (**B**). **F**) Significance of the indicated TFBS motif associated with *TEAD* in D0, D4, and D6 enhancer regions. A representative factor from each family of TFs is shown. **G**) Dot plot indicating significance of the indicated interrogated motif in the FOX family of TFs at either D0 (light teal), D4 (medium teal), or D6 (dark teal). Size of dot is reflective of RPKM expression level at the indicated day. RPKMs are an average of all three donors profiled by RNAseq. **H**) Stacked histogram of factors whose motifs were associated with the FOX family member *FOXP1*. TF families are colored according to conserved binding sequence. I) Significance of the indicated TFBS motif associated with *FOXP1* in D0, D4, and D6 enhancer regions. A representative factor from each family of TFs is shown

To examine more closely our observation that families of TFs were shifting their associations during differentiation, we isolated three groups of interest in the network analysis. First, we isolated the AP1/MAF family of TFs which had the highest overall significance at each day, and were located centrally in the network analysis. AP1/MAF family members share a core GATxxxTCA motif, however specificity for flanking and intervening sequences around these core nucleotides varies widely within the TF family. Plotting motif significance within enhancers at each timepoint of AEC differentiation against their overall expression level allowed us to visualize how their enrichments were changing over time in culture (Fig. 4A). We observed that *FOS* and *ATF3* were the most highly expressed AP1/MAF family members at D0, with decreased expression on D4 and D6. In contrast, *FOSL2* and to a lesser extent *JUNB* expression increased over differentiation while simultaneously increasing in motif significance. For the rest of the AP1/MAF family, expression was either negligible (ex., *BACH2*), or despite expression changes during differentiation their motif enrichment remained relatively constant (ex., *NFE2L2*, *MAFB*, *MAFF*).

To examine how TF interactions were changing with AP1/MAF family members, we selected FOS as a representative TF from this group and plotted the number of all of the TFs predicted to interact with this factor on each day of differentiation (Fig. 4B). Significance of associated motif enrichment cut-offs from Fig. 3 were maintained for this analysis (D0: p < e^− 50^, D4: p < e^− 100^, D6 p < e^− 100^). We observed that the overall number of significant interactions with FOS decreased over the course of 2D AEC differentiation. We also observed that, while individual members of a given TF family were changing, many of the predicted interactions within a family of TFs were maintained. For example, FOS was predicted to interact with ETS, bHLH, FOX, Homeobox, TEAD, SP1/KLF, HNF1, and STAT family members on each day of differentiation, but the total number of those interactions varied on each day. In contrast, FOS interactions with NFkB were only significant on D0, and FOS interactions with CP2 and NKX family members were only significant on D4 and D6.

To further illustrate these differential motif association dynamics, we plotted the significance of motif enrichment by days in culture for a representative from each of the top 10 families associated with FOS (Fig. 4C). We observed that NFkB-p65 (*NFKB2*) association with *FOS* decreased over days in cultures, while other motifs generally increased with time, the most striking of which was association with *PBX2*, a homeobox domain TF that is expressed in human AEC and in primary human alveolar cells in the IPF Cell Atlas [70], but has not been previously characterized during AEC differentiation.

We repeated the above analysis for the TEAD TF family, which has recently been reported to influence AEC differentiation and maintenance of AT1 cell identity [13, 71, 72]. TEAD has 4 family members, all of which are expressed in the 2D AEC model of differentiation. Plotting expression against motif enrichment revealed that *TEAD2* expression increased concomitant with motif enrichment during AEC differentiation, and that motif enrichment varied dramatically among family members (Fig. 4D). The number of significantly associated motifs increased during AEC differentiation (Fig. 4E), with D4 and D6 gaining interaction with *NKX*, homeodomain, *HSF*, and *SMAD* family members. An example of this was NFKB-p65 (*NFKB2*) association with *TEAD*, which was predicted to increase dramatically during AEC differentiation (Fig. 4F). However, interactions with *AP1/MAF*, *FOX*, and *STAT*, and *ETS* family members remained unchanged throughout, indicating that while some predicted associated motifs varied during differentiation, others remained constant. Other known factors that are known to be critical to AEC fate, *NKX2–1* and *FOXA1*, were also predicted to increase interactions with *TEAD*.

We then isolated a few key members of the FOX family to study their associated motif interactions during AEC differentiation. FOXA1 and FOXA2 are key transcriptional regulators of alveologenesis in the lung [67, 73–75], and FOXO/FOXP factors also have known roles in maintenance of AEC cell fate [76–78]. We observed that despite having a conserved consensus core sequence (xGTTTAxx) family member motif enrichment varied dramatically (Fig. 4G). Overall there were slight decreases in significance for *FOXA1* and *FOXO3* motif enrichment by days in culture, despite increasing expression of these factors, whereas *FOXP1* motif enrichment increased dramatically even though there was no change in RNA expression. The number of interactions with *FOXP1* also increased dramatically during AEC differentiation (Fig. 4H). *FOXP1* interactions with *SOX* family members were observed on all days, however D4 and D6 also had predicted interactions with *GATA*, *AP1/MAF*, homeobox, and ZF family members. While *HOXB13* was the factor with predicted highest associated motif significance (Fig. 4I), we did not observed expression of HOXB13 in 2D AEC RNAseq. This may indicate that other Homeobox family members with similar core binding sequences are responsible for this signature. We also observed increased association between *FOXP1* and *SOX4* on D4 and D6. *SOX4* RNA is expressed in 2D AEC RNAseq and SOX family members play key roles in lung and alveolar differentiation, indicating that this interaction may be of importance to AEC differentiation.

Our observations that (1) FOX family members were represented in the FAIRE open chromatin regions of AT2 and AT1-like cells, (2) FOX family member binding dynamics were likely altered during 2D AEC differentiation within enhancers, (3) there was a high degree of interconnectivity between FOX family members and other transcription factor networks, (4) FOX family members, in particular FOXA1, had changes in transcriptional levels during AEC differentiation, and (5) the known role of FOXA1 in alveologenesis [67], focused our attention on the interactions of FOXA1 with other TFs to facilitate alveolar differentiation.

### FOXA1 binding in AEC is associated with TF networks in an AEC cell type-specific manner

**Fig. 5:**
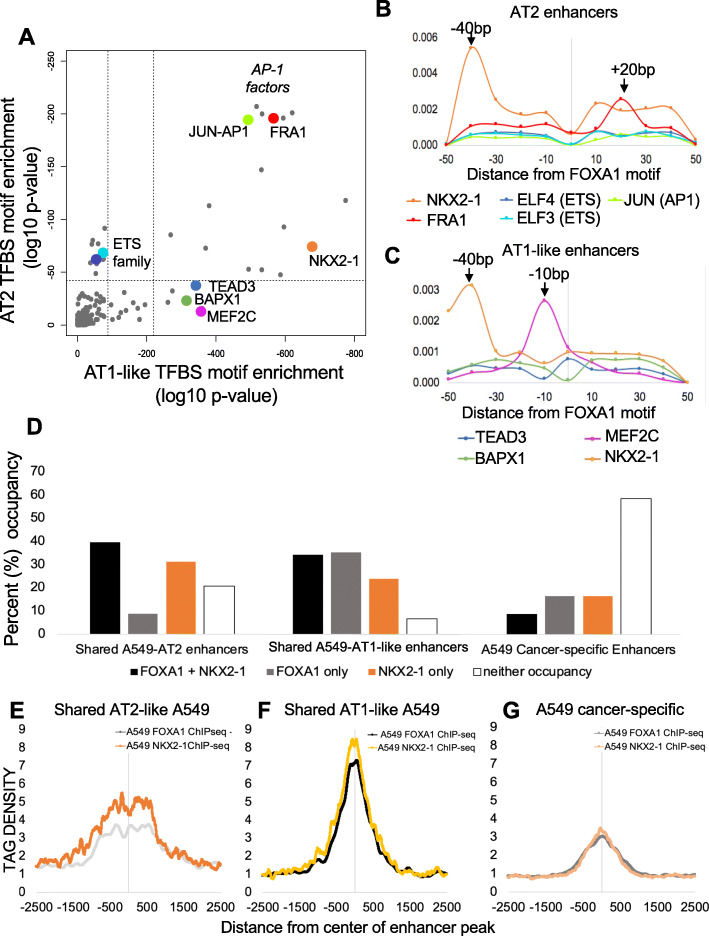
FOXA1 binding in AEC is associated with TF networks in an AEC cell-type specific manner. **A**) HOMER-computed TFBS enrichment for Associated Motifs surrounding the *FOXA1* predicted binding motifs in AT1-like (x axis) and AT2 (y axis) cell-specific enhancers. Dotted line indicates threshold for statistical significance. **B**) Predicted binding site distance of the several statistically significantly enriched TFBS motifs from center of *FOXA1* binding motif for AT2-cell enhancers. Zero position is the center of the *FOXA1* predicted binding site. Y axis = density of predicted Associated Motif(s) at indicated bp distance from center of *FOXA1* motif. **C**) Predicted binding site distances of the indicated TFBS motifs from center of *FOXA1* Interrogated Motif for the AT1-like cell enhancers. **D**) Distribution of FOXA1 and NKX2–1 occupancy of A549 enhancers that are also present in AT2 cells, AT1-like cells, or are specific to the cancer phenotype. **E**-**G**) ChIP-seq of FOXA1 and NKX2–1 in A549 lung cancer cells. Tag density of ChIP-seq reads plotted relative to the center of the enhancer peak. Tag densities between ChIP-seq runs are normalized per millions mapped. **E**) A549 enhancer peaks that had > 50% overlap with AT2 cell enhancer regions, **F**) A549 enhancer peaks that had > 50% overlap with AT1-like enhancer regions, **G**) A549 enhancers without overlap to AEC enhancers

Our work correlating epigenetic alterations with gene expression changes revealed that FOXA1 was expressed in both AT2 and AT1-like cells, was upregulated during AEC differentiation, and showed motif enrichment at the center of FAIRE-labeled open regions in both cell types. It is known that FOXA1 can translate epigenetic signatures into enhancer driven lineage-specific transcriptional patterns by acting as a pioneering transcription factor to open chromatin and coordinate cellular differentiation [79, 80]. Therefore, we decided to study the predicted binding behavior of FOXA1 in relation to other TFBS motifs within enhancers. The typical nucleotide spacing of TFs bound together in a heterodimeric complex is between 1 and 50 bp depending on the factors involved [63–65]. To further characterize which of the identified TFs might associate with FOXA1 to maintain AT2 cellular identity or redirect FOXA1 to alternate enhancers to promote AT1-like differentiation, we gathered +/− 50 bp from the predicted FOXA1 binding site within cell-type specific enhancers and re-ran the HOMER motif analysis, excluding FOXA1 as it was a criterion for sequence selection. We found that in AT2 cells, FOXA1 motifs co-occur alongside ETS family member motifs with high statistical significance (Fig. 5A). This predicted association shifts in AT1-like cells, where TEAD family members and MEF2C are highly significantly enriched motifs alongside FOXA1. Consistent with these observations, we saw a decrease in ETS1 expression and increase in MEF2C during AEC differentiation, providing a possible mechanism for FOXA1 transcriptional heterodimers based on relative expression levels of cofactors. In addition, we observed enrichment of NKX2–1 and NFI in proximity of both AT2 and AT1-like FOXA1 predicted motifs.

It is known that FOXA1 can associate with several TFs including NFI to direct cellular differentiation [81]. Further, the physical interaction between FOXA1 and NKX2–1 has been observed previously in AEC [82], bolstering confidence that our analysis is identifying transcription factor complex interactions that influence epigenetic enhancer state alterations during AEC differentiation. To further characterize this relationship, we analyzed the distance from FOXA1 motifs to enriched TFBS in AT2 cells (Fig. 5B) and AT1-like cells (Fig. 5C). Strikingly, we observed a high degree of enrichment for NKX2–1 motifs − 40 bp away from the predicted FOXA1 binding motif in both AT2 and AT1-like cells, which could indicate an interactive relationship between the two throughout AEC differentiation as previously reported [82]. In addition, we observed enrichment of the FRA1/FOSL1 motif at the + 20 bp position from the FOXA1 motif in AT2 cells, as well as a high level of enrichment for the MEF2C motif at the − 10 bp position in AT1-like cells. In concordance with previous reports, these findings strongly indicate FOXA1 may partner with multiple transcription factors to facilitate AEC differentiation.

To determine if motif prediction was representative of actual TF factor binding patterns within enhancers, we reanalyzed publicly available ChIP-seq data that was generated in A549 cells, a cancer cell line derived from lung adenocarcinoma. AT2 cells have been thoroughly studied as a cell population that can give rise to lung adenocarcinoma [83–85]. While using a lung cancer cell line model is not ideal for studying differentiation of normal human cells, it is the only publicly available model of human lung origin where ChIPseq for FOXA1, NKX2–1, and both enhancer marks H3K27Ac and H3K4me1 have been generated with high enough quality for downstream analysis [86, 87]. We defined enhancers in A549 cells using the same criteria as in AEC, namely > 50% overlap of H3K27Ac and H3K4me1 peaks. The epigenome is known to be heavily dysregulated during the carcinogenic process, so we further subclassified enhancers in A549 cells by > 50% peak overlap with our previously defined AEC enhancers. This resulted in enhancers of three categories: A549 enhancers that were also present in AT2 cells (1500 regions, 5.2% of total A549 enhancers); A549 enhancers that were also present in AT1-like cells (9678 regions, 32.8% of A549 enhancers); and A549 enhancers that were uniquely present in the cancerous cell line (18,303 regions, 62% of A549 enhancers) and may therefore may represent dysregulated enhancer activity. We did not detect discernable differences in TFBS motif enrichment between subsets of A549 enhancers based on their overlap with enhancers in AT2 and AT1-like cells (**Fig. S****6**). To determine TF occupancy within these categories of enhancers, we reanalyzed ChIP-seq data for endogenous FOXA1 originally generated by the ENCODE Consortium [88], and a separate study that determined occupancy for ectopically expressed NKX2–1 in A549 cells [89]. Unfortunately, publicly available MEF2C and FRA1/FOSL1 ChIP-seq datasets were not available in lung-derived cell lines.

Overall, only 13.9% of A549 cell enhancers exhibited co-occupancy of FOXA1 and NKX2–1 by ChIP-seq. However, we observed differences in co-occurrence from this average depending on whether the A549 enhancer was categorized as ‘shared with AT2’, ‘shared with AT1-like’, or ‘A549 cancer-specific’. For shared A549-AT2 enhancers, 39.6% had co-occupancy of NKX2–1 and FOXA1 (Fig. 5D). Similarly, NKX2–1 and FOXA1 peaks were co-occurrent in 34.3% of A549-AT1 enhancers. In contrast, A549 cancer-specific enhancers contained considerably fewer instances of FOXA1 and NKX2–1 peak co-occurrence (8.6%). Together this indicated that co-occupancy of FOXA1 and NKX2–1 within “normal” AEC enhancers occurred approximately three times more often than within A549 cancer-specific enhancers. Indeed, almost 60% of cancer-specific A549 enhancers lacked any binding for FOXA1 or NKX2–1 (Fig. 5D), suggesting that the colocalization of FOXA1 and NKX2–1 observed in A549 cells is primarily driven by enhancers preserved in normal tissues.

To determine the relative positioning of FOXA1 and NKX2–1 in the cell type-specific subsets of enhancers, we extracted sequence alignment map (SAM)-level data and used HOMER to generate Tag densities at the cell type-specific peak regions. In AT2 cell-type enhancers that are also present in A549 cells, FOXA1 and NKX2–1 exhibited enrichment that was spread across the central 500 bp of the enhancer peaks (Fig. 5E). In contrast, AT1-like cell enhancers also present in A549 cells showed a high degree of enrichment for both factors toward the central 100 bp of the enhancer peaks. Based on known binding dynamics for TFs within enhancers, we would expect TFs involved in activation and maintenance of the active enhancer to be clustered toward the center of the enhancer region (Fig. 5F). In cancer-specific enhancers in A549 cells, there was far less enrichment for both NKX2–1 and FOXA1, with no obvious differences in TF position relative to the center of the peak (Fig. 5G). Intriguingly, the NKX2–1 and FOXA1 datasets both exhibited a dip at the exact center of the peak for AEC-shared enhancers, which may be due to the presence of another factor. To investigate what factor might be bound there, we extracted the central 100 bp from those AT1-like enhancers shared with A549 cells that also were co-occupied by NKX2–1 and FOXA1. JunB (*p* = 3.2 × 10^− 16^) and MEF2C (*p* = 1.4 × 10^− 8^) were the predicted factors to bind this center-of-the-peak region. This could indicate that FOXA1 and NKX2–1 operate in a trimeric complex with either MEF2C or AP1/JunB family members.

### Identification of NKX2–1 and MEF2C as FOXA1-associated TFs that specify lung epithelium differentiation

Once we had characterized the relationships between TFBS motif enrichment and epigenetic state alterations during AEC differentiation, we sought to determine if the predicted interactions were unique to lung differentiation or a common phenomenon shared among other cell lineages. To investigate this, we utilized publicly available high-quality ChIP-seq datasets from normal tissues profiled by the ROADMAP epigenomics project (76 samples) and ENCODE (6 samples) [88, 90]. To define what an enhancer was across multiple tissue types, we used the criterion that each cell type needed to have high-quality ChIP-seq data for H3K27Ac and H3K4me1. The H3K27Ac peaks in each cell type were then filtered to include only those that had > 50% overlap with H3K4me1 peaks in the same cell type.

**Fig. 6:**
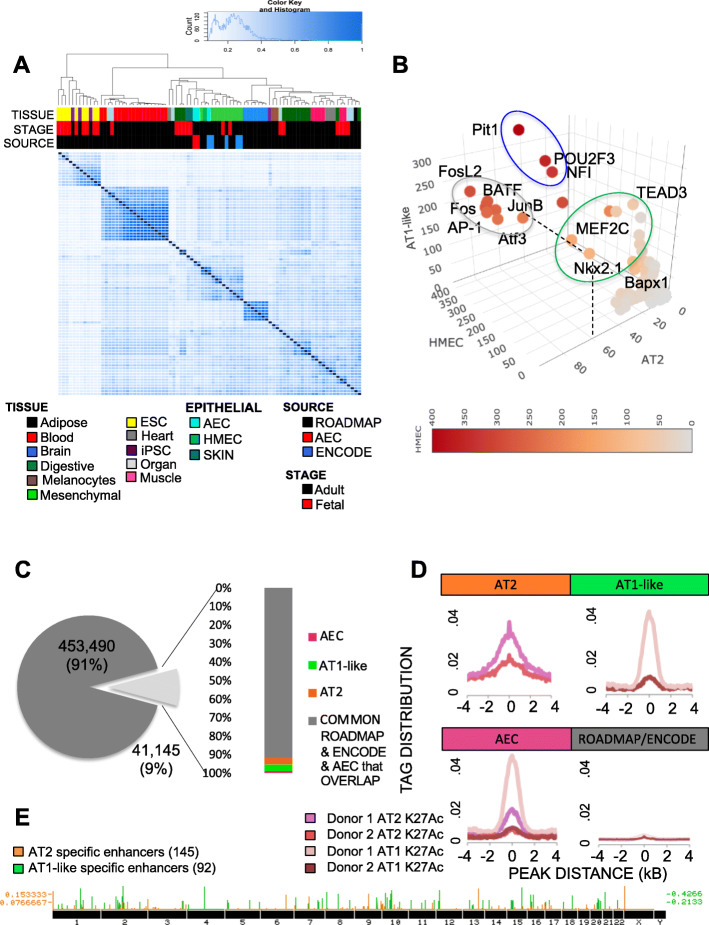
Comparative analysis of AEC enhancers with ROADMAP and ENCODE enhance-ome reveals 237 alveolar epithelial-specific enhancers. **A**) Diffbind plotting of similarity between enhancer regions. Tissue = ROADMAP or ENCODE indicated cell type. Stage = age of donor, subdivided into pre- and post- natal. Source = origin of the data used in the analysis. **B**) Three-dimensional scatterplot of enrichment for each of the TFBS present in HOMER in AT2, AT1-like, and HMEC enhancers. Red scale coloring indicates level of enrichment in HMEC. Grey circle indicates TFBS motifs enriched in all 3 cell types. Blue circle indicates TFBS enriched in HMEC and AT1-like cells, green circle indicates TFBS motifs enriched specifically in AT2 and AT1-like cells. **C**) Pie chart indicating similarity between ROADMAP/ENCODE enhancers and AEC-identified enhancers. AEC = regions labeled enhancers in both AT2 and AT1-like cells but not in any ROADMAP or ENCODE dataset. **D**) Histogram indicating tag-density of enhancer-specific enrichment for H3K27Ac between biological replicates. **E**) Distribution of AT2 and AT1-like cell-specific enhancer regions genome-wide

Diffbind analysis showed clustering of embryonic stem (ES)/induced pluripotent stem (iPS) cells as distinct from all other cell types (Fig. 6A). Hematopoietic lineages also clustered separately from other tissues (including purified blood cell types, thymus and spleen). Interestingly, epithelial (light blue) and mesenchymal (light green) cell types were more similar to each other than all other cell types examined, with AECs closely related to the epithelial datasets present, which were human mammary epithelial cells (HMEC) and foreskin. We saw slight variation in the cell types most associated with AEC when clustered by H3K27Ac or H3K4me1 marks individually (**Fig. S****7**); however, breast epithelium was consistently one of the most closely associated tissues by epigenetic signatures that were available from ROADMAP and ENCODE, which may be due to overall underrepresentation of epithelial tissues in these databases.

However, breast and lung both undergo branching morphogenesis, and FOXA1 has a demonstrated role for both tissues in this process [67, 91]. Therefore, to explore the similarities and differences between these tissues, we wanted to determine if the transcriptional co-network of TFs associated with *FOXA1* was common to both or if instead, FOXA1 transcriptional networks varied between these tissues. To do so, we evaluated motif enrichment within 50 bp of *FOXA1* predicted sites in primary human mammary epithelial cell (HMEC) enhancers, repeating the process that was undertaken in Fig. 3A. In total, 45% of AEC enhancer regions overlapped with HMEC enhancers, suggesting that although HMECs were the most closely related cell type studied, there was still considerable variation between their epigenetic states. 53% of all enhancer peaks in HMECs contained the predicted FOXA1 binding motif. Motif enrichment analysis was then re-run on the 100 bp surrounding the predicted *FOXA1* binding sites in HMEC enhancers. Because enhancer regions were selected based on the presence of *FOXA1*, FOX family motifs with similar sequence to *FOXA1* were eliminated from the subsequent analysis. A three-dimensional scatter diagram of enrichment measurements for all available TFBS motifs in AT2, AT1-like, and HMEC enhancer regions was performed to visualize similarities and differences between these three cell types (Fig. 6B). Enrichment for *FOS/JUN* (AP-1) motifs was observed in proximity to *FOXA1* in all three tested cell types (grey circle), indicating that partnering between FOXA1 and FOS/JUN factors may play a conserved role in epithelial cell types. A separate cluster of TFBS motifs enriched in AT1-like cells and HMECs also emerged (blue circle), which included *PIT1*, *POU2F3*, and *NF1*. NF1 is a known binding partner of FOXA1, whereas POU2F3 and PIT1 are involved in cellular fate determination. This could be reflective of the role these factors play in cellular differentiation [92–94]. Lastly, a separate cluster of TFBS enriched in AT2 and AT1-like cells but not HMECs was observed (green circle). This included *NKX2–1*, a known lung-specific lineage factor that is critical for lung specification, as well as *TEAD* family members and *MEF2C*. Therefore, we have identified a high confidence set of transcription factors that appear to act in concert to coordinate AEC differentiation in vitro and distinguish between lung and breast enhancer identity.

### Identification of AT2 and AT1-like enhancers unique to AEC from the known compendium of human enhancers

To determine how the transcription factor coregulatory networks described above work in concert to specifically activate cell type specific enhancers, we first identified enhancer regions that were present only within AEC. The considerable variation in enhancer location across all tissues present in ROADMAP/ENCODE and the observation that enhancer regions best recapitulated the epigenetic signature of differentiating AECs gave rise to the idea that we could utilize publicly available datasets on enhancer locations to define AEC cell-specific enhancer signatures for both AT2 and AT1-like cells. To do so, the entire complement of ROADMAP [90] and ENCODE [88] enhancers for the 82 normal cell types across many organ types was merged to create one master list containing all regions within the human genome identified as enhancers, which we will refer to as the “enhance-ome”. Cancer-derived enhancer signatures were omitted due to their potential perturbation by the carcinogenic process. The locations of AT2 and AT1-like cell enhancer regions were then compared to the enhance-ome. AECs had 41,145 active enhancers at 9% of all identified normal enhance-ome regions (Fig. 6C). Of those 41,145 sites in AECs, 92% were also considered enhancers in ROADMAP and ENCODE data sets, providing us with a high level of confidence that our AEC-defined enhancers were consistent with observations from other sources. Within the enhancers present in AEC but not in ROADMAP or ENCODE, 295 enhancer regions were active in both AT2 and AT1-like cells (termed AEC), 1277 enhancer regions were only active in AT2 cells (ie., not present in AT1-like, ROADMAP or ENCODE), and 1706 enhancer regions were only present in AT1-like cells.

To validate these regions as either AT2 or AT1-like cell-specific enhancers, we utilized the biological replicate ChIP-seq data from Donor 2. H3K27Ac peak enrichment was centered similarly between Donor 1 and Donor 2 in both AT2 and AT1-like samples (Fig. 6D); however, the overall enrichment was lower for the biological replicate from Donor 2. Subsetting the AT2 cell-specific and AT1-like cell-specific peaks from Donor 1 to overlap with peaks called from Donor 2 resulted in identification of 145 AT2 cell-specific and 92 AT1-like cell-specific high-confidence enhancers (Fig. 6E). In addition, we also sought to characterize associations between enhancers and target gene expression, often called “enhancer-gene pairs”. To do so, we utilized both nearest-neighbor (**Fig. S****8****A**), and GTEx-annotated SNP-gene associations where SNPs were located within enhancers (**Fig. S****8****B**). Nearest-neighbor analysis included all AT2 and AT1-like enhancers and loss or gain of a nearby enhancer trended toward respective changes in nearby gene expression, though many exceptions existed. In contrast, GTEx-associated enhancer-gene pairs were limited by the necessity of having a SNP located within the enhancer. Of the 145 AT2 cell-specific enhancers, 61 (42%) had SNPs located within them. Of the 92 AT1 cell-like specific enhancers, 77 (84%) had SNPs located within them and several had multiple SNPs per peak. GTEx recognized 69% of AT2 cell enhancer regions containing rsIDs and 76% of AT1-like cell enhancer region containing rsIDs. Of those, 16 SNPs in AT2 cells and 125 SNPs in AT1-like cells were significantly correlated with alterations in gene expression for at least one gene in lung tissue. Again, multiple SNPs within the same or nearby enhancer regions were functionally linked to the same gene or multiple genes; therefore, we identified 19 AT2 cell specific enhancer-gene pairs and 54 AT1-like enhancer-gene pairs utilizing GTEX, for a total of 73 AEC enhancer-gene pairs (Supplemental Table 2). In general, most enhancer-gene pairs based on GTEx SNPs did not show changes in gene expression during AEC differentiation (Figure S8B). Only 19 genes from the GTEX enhancer-gene pair analysis had significantly altered expression during AEC differentiation with log2 fold changes in gene expression that matched the direction of enhancer activity (Supplemental Table 3). Ranking the enhancer-gene pairs that occurred using both methods, the top enhancer-gene pair in AT2 cells was at the surfactant protein A1 (*SFTPA1*) locus, a known AT2 cell-specific gene (Figure S9, left). The top AT1-like cell type specific enhancer identified was linked to aminoadipic semialdehyde synthase (*AASS*), which catalyzes lysine degradation. Lysine is an essential amino acid required for protein production and synthesis in lung is thought to be downregulated to confer partial resistance to viral infections [95]. Since AT1 cells comprise the majority of the epithelial surface, they likely play an important role in viral immunity.

### MEF2C:FOXA1:NKX2–1 transcription factor heterotrimeric complexes are enriched in AT1-like cell type specific enhancers

**Fig. 7:**
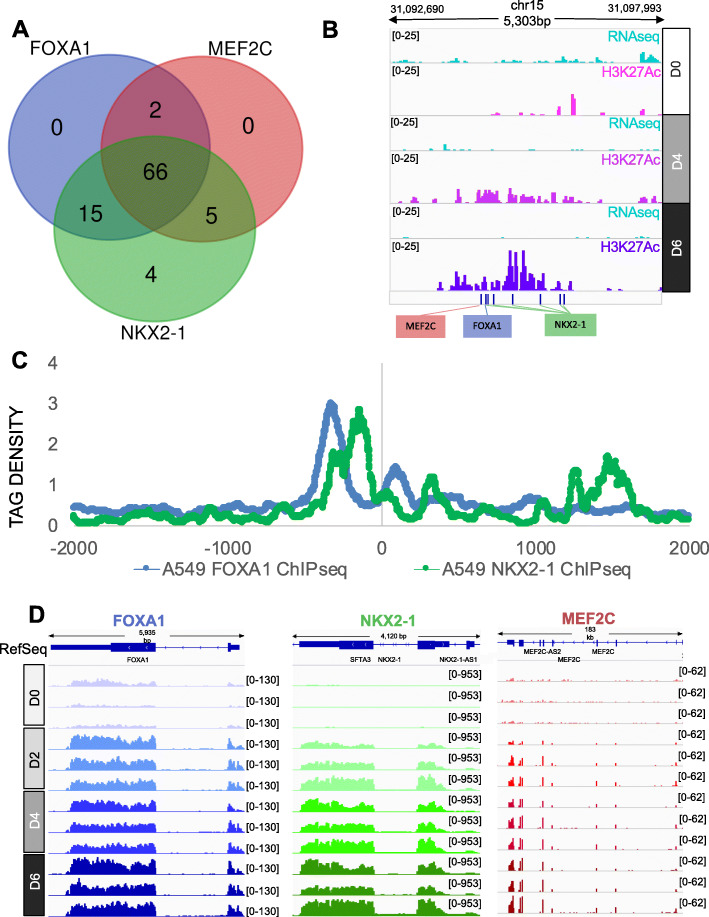
MEF2C:FOXA1:NKX2–1 transcription factor heterotrimeric complexes are enriched in AT1-like cell type specific enhancers. **A**) Presence of predicted NKX2–1, MEF2C, and FOXA1 binding sites within the 92 AT1-like cell-specific enhancer regions. **B**) Example of one specific locus with an AT1-like cell type specific enhancer and the relative positioning of predicted *FOXA1*, *NKX2–1*, and TFBS. **C**) A549 cell line ChIP-seq of FOXA1 (blue) and NKX2–1 (green) distribution in AT1-like cell-specific enhancers. Tag Densities are centered on the middle of the cell type specific enhancer peak. **D**) IGV display of FOXA1 (blue), NKX2–1 (green), and MEF2C (red) expression levels during AEC differentiation. D0 = Day 0 (AT2), D2 = Day 2 (AT1.5, intermediate), D4 = D4 (AT1.5, intermediate), D6 = Day 6 (AT1-like)

Although we identified transcription factor co-regulatory networks as well as AEC cell-type specific enhancer regions, the influence of the *FOXA1*-associated TFBS on cell type-specific enhancer regions remained unanswered. To address this, we analyzed the distribution of TFBS motifs within the AT2 and AT1-like cell-type specific enhancers and found that all of the AT1-like cell-type specific enhancers had motifs for at least one of the TFs that were identified as associated with *FOXA1* in AT1-like cells. The majority of AT1-like cell-specific enhancers had predicted motifs for all three TFs: *FOXA1*, *NKX2–1*, and *MEF2C* (Fig. 7A). Many of the AT1-like cell-specific enhancers had predicted motif distributions consistent with the TF spacing we observed previously, consistent with what is known about the interaction of these TFs in the literature (Fig. 7B)**.** To determine the relationship between FOXA1 and NKX2–1 positioning in AT1-like cell type-specific enhancers, relative FOXA1 and NKX2–1 ChIP-seq tag density enrichment was plotted across the 92 AT1-like cell-specific enhancers from the publicly available A549 datasets (Fig. 7C). We observed that staggered spacing between FOXA1 and NKX2–1 peak summits offset was larger in the ChIP-seq data than from motif prediction (190 bp in ChIP-seq vs 40 bp in motif prediction), which may be due to a loss of resolution in the ChIP-seq due to fragmentation size of the ChIP libraries. To determine if expression levels may in part explain the presence of these factors at AT1-specific enhancer sites, we plotted RNAseq reads for each of the three identified TFs that were enriched in AT1-like (D6) enhancer peaks, *FOXA1*, *NKX2–1* and *MEF2C*, as a function of time (Fig. 6D). We observed that, while *FOXA1* and *NKX2–1* are expressed in AT2 (D0) cells, their expression increases dramatically during 2D AEC differentiation (Fig. 7D). *MEF2C* expression in AT2 cells is negligible, and similarly increases expression throughout days in culture. Indeed, relatively higher levels of *NKX2–1* have been observed previously in AT1 cells vs. AT2 cells in the IPF Cell Atlas (**Fig. S****10**, 97). Previous studies in mice have also established a critical role for NKX2–1 in maintenance of AT1 cell fate [37, 96]. Our results suggest the association of FOXA1, NKX2–1, and MEF2C may act in a cooperative heterotrimeric TF complex which binds to AT1-like enhancers as part of a coordinated effort to differentiate the alveolar epithelium, which is reflected in concomitant alterations to the epigenetic state to mediate cellular fate determination. While this heterotrimeric complex may be important for AEC differentiation, it is but one of many interactions that occur among over a dozen families of transcription factors to facilitate this process.

## Discussion

We set out to characterize epigenomic alterations that occur during AEC differentiation and how they influence cellular identity. We found that the enhancer-associated epigenetic signatures of FAIRE open regions, H3K27Ac and H3K4me1 peaks were most closely associated with changes in gene expression during AEC differentiation. Exploring this linkage further, we found that the composition of predicted TFBS motifs changed dramatically during in vitro AT2 to AT1-like cell differentiation, with some TFs (e.g., FOS, ETS1, and NKFB1) enriched in AT2 cell maintenance losing expression and simultaneously having decreased predicted binding to enhancer regions in AT1-like cells. In addition to TEAD family members [13, 72], we also found that others, including *MEF2C*, increased in expression and had corresponding increases in predicted TF binding when transitioning to an AT1-like cell fate. In contrast, there were several TFs (e.g., SNAIL, TWIST, GLI family members, and several but not all ZNF family members) whose predicted binding did not appreciably change in either FAIRE open regions or enhancers during differentiation.

We also determined that the TF FOXA1, which acts as a pioneering transcription factor and is known to regulate branching morphogenesis of the lung and AT2 cell maintenance, may play a critical role in human AT2 to AT1 cell differentiation by partnering with the lung-specific TF NKX2–1 to open chromatin and facilitate AEC differentiation. This may be accomplished by switching TF heterotrimeric complex members during differentiation, as we observed differential enrichment for FRA1/FOSL1 and MEF2C in AT2 and AT1-like cells, respectively. This heterotrimeric complex member switching could facilitate alternate enhancer target localization or alter the function of the complex. Interestingly, the previously reported NKX2–1:FOXA1 interaction at the SFTPC promoter was deemed inhibitory, turning off SFTPC expression in AT2 cells [82], whereas we observe these predicted interactions in regions bearing epigenetic marks characteristic of “active enhancers” associated with transcriptional activation. Additionally, it has been previously reported that loss of NKX2–1 can direct the FOXA1/FOXA2 TF axis to alter cell fate from lung to stomach phenotypes [97], specifically for AT1 cells [37]. Our analysis provides a basis for connecting these disparate lines of evidence: namely, that beyond the known role of NKX2–1 in establishment of the lung endodermal lineage from thyroid [98, 99], and the role of FOXA1 in lung branching morphogenesis [67], the FOXA1:NKX2–1 interaction may be pivotal in regulation of epigenomic fate during AEC differentiation.

*TEAD* was identified as one of the most enriched motifs in FAIRE peaks in AT1-like cells, and within enhancers TEAD interactions with other TFs increased during AEC differentiation to include SMAD, NKX2–1, and HSF factors, while retaining interactions with AP1, FOX, ETS, and STAT family members throughout. These findings are consistent with several recent publications in both mouse and human indicating a role for YAP signaling in driving AEC differentiation [13, 72]. However, our results indicate that the interactions that determine cell fate during AEC differentiation are likely more complex than a single TF or TF interaction, and rather involve a shift in an entire network of TFs and enhancer activation in AT2 vs AT1 cells.

Interestingly, PIANO analysis revealed that FAIRE gain was also significantly associated with downregulation of associated gene expression, perhaps as a result of repressor factor occupancy at sites outside of active enhancer regions. Indeed, we see differences in identity, significance, percent peak occupancy, and distribution of TFBS motifs between FAIRE and enhancer regions, indicating these data types are not completely synonymous. While determining the precise functional role for any of the TFs we have uncovered in this study will require further in vitro characterization, we provide here a compendium of highest-priority TF candidates that recapitulate on a genome-wide scale our previous in vitro findings that were determined at individual loci.

We also investigated conservation and uniqueness of AT2 and AT1-like TF co-regulatory networks by examining the significance of individual TFBS motif enrichment in the most closely related cell type profiled by the ROADMAP and ENCODE databases, that of human mammary epithelial cells (HMEC). We discovered a subset of FOXA1-associated TFs common to all three cell types including NKX2–1, and to a lesser extent MEF2C and TEAD3, that were enriched in AECs as compared to HMECs. In contrast, enrichment for *FRA1/FOSL1* was observed at + 20 bp downstream of the *FOXA1* motif. Therefore, FRA1/FOSL1 interaction with FOXA1 may play a critical role in multiple organs. Follow-up work in mouse models will be important to determine if conditional knockout of FRA1/FOSL1 is able to recapitulate the deleterious effects on branching morphogenesis seen in FOXA1/FOXA2 double knockout mice [67].

It should be noted that members within the same family of transcription factors often have nearly identical TFBS motifs. Throughout the paper, we refer to specific TFBS as enriched based on HOMER motif predictions; however, in the absence of confirmatory ChIP-seq (DNA occupancy) and RNA-seq (expression) data, these motifs could be bound by any one or multiple TF family members with similar DNA binding preferences. For example, the FOXA motif is nearly identical for all three FOXA family members (FOXA1, FOXA2, and FOXA3), limiting occupancy predictions based purely on motif enrichment to a family of related TFs rather than implicating a specific TF. Complicating the interpretation of FOXA motif enrichment is their known compensatory roles in branching morphogenesis of the lung and alveolar epithelial differentiation [67]. Another is the known role of Etv5 in AT2 cell fate maintenance [66]. Etv5 is a member of the ETS family of TFs, and while there is no specific entry for Etv5 in HOMER, the high levels of enrichment for ETS family members during AEC differentiation and their shared TFBS recognition is consistent with the known role of this ETS family member in alveolar fate determination.

We also identified a high-confidence set of AEC cell type-specific enhancers that were present in biological replicates of AT2 or AT1-like cells, but not present in other ROADMAP or ENCODE normal tissue databases. We found that key transcription factor co-regulatory network partners identified in our genome-wide analysis were also present at highly selective AT1-like specific enhancer sites, but there were relatively few cell-type specific enhancers that were only present in AT2 or AT1-like cells. This would support the notion that there is no one specific TF that drives AT1-like enhancer activation but is instead the result of combinatorial TF activity that is acting broadly across the genome.

## Conclusions

In summary, we have identified epigenetic signatures characteristic of primary human alveolar epithelium and have elucidated mechanistic insights into how this shifts in an in vitro model of primary AT2 to AT1-like cellular differentiation. These epigenetic signatures are being made publicly available to further understanding of the alveolar epithelial cell differentiation process with particular emphasis on how epigenetic signatures dictate the coordinated pathways that result in altered cellular fate [7, 35]. AEC differentiation in vitro from purified adult human, rat, and mouse AT2 cells is considered a model of wound healing [100, 101], as adult AT1 cells must be replenished after exposure to and damage from a slew of particulate and chemical insults present in the air we breathe [102]. The ability of the TFs we have identified to facilitate this process may be affected by these environmental insults, leading to disrupted AEC differentiation and wound healing. This can in turn manifest as diseases of the distal alveolar epithelium, such as IPF, COPD, and lung adenocarcinoma (LUAD). Importantly, many of the transcription factors we have identified in this study have known roles in these disease processes. Specifically, FOXA1 plays a significant role in non-small cell lung cancer [103], MEF2 family members have a role in lung carcinoma [104, 105], TEAD family members have known roles in carcinogenesis of epithelial tissues [106], and NKX2–1 has a long history of involvement in lung cancer, COPD and IPF [87, 107–110]. Understanding the relationship between disruption of the epigenetic state during AEC differentiation and the development of lung diseases could open up an entirely new avenue of therapeutic options for these often-fatal diseases.

## Materials & methods

### Isolation and culture of human alveolar epithelial cells

Donor lungs were obtained through the IIAM tissue procurement network, which provide non-transplantable human organs and tissues for medical research. Lungs were processed within 3 days of death. AT2 cells were isolated from cadaveric human lungs that were declined for donor transplantation. Donor 1 was a 62-year-old Caucasian male. Donor 2 was a 25-year-old Caucasian male. Donor 3 (used for RNA only) was a 73-year-old Caucasian female. None of the donors died from lung-related injury or complications. We selected the lobe of the lung that had no obvious consolidation or hemorrhage by gross inspection. The lung tissue processing protocol was modified from previously published reports [8, 111] with the following modifications: Enhanced selection of epithelial cells was performed by using CD326 (EpCAM) beads (Miltenyi Biotec #130–061-101) and rotating the cells for 10 min at 4 °C followed by 20 min at RT. Cells were collected by magnetic selection using LS columns (Miltenyi Biotec #130–042-401). AT2 cell purity was determined by staining of cytospin preparation with NKX2–1 (1:100, Leica Biosystems, Cat # NCL-1-TTF1). Donor 1 was 83% positive, Donor 2 was 96% positive, and Donor 3 was 79% positive, however, not enough cells were collected for chromatin profiling of this third lung, so the sample was used for RNA-seq only. For harvesting chromatin, AT2 cells were plated at 4 × 10^6^ cells per well in 6-well Corning plates (Primaria) and incubated at 37 °C in 50:50 DMEM high glucose: DME-F12 media supplemented with 10% fetal bovine serum (FBS). For harvesting of DNA and RNA, AT2 cells were seeded on collagen-coated transwell-COL inserts (Corning, #3493) at a density of 1 × 10^6^/filter. 1 million cells were used for DNA and RNA extraction, 2–5 million cells were used for chromatin immunoprecipitation (ChIP-seq) of histone marks, and 10 million cells were used for CTCF ChIP-seq.

### Western blotting

Western blots were performed as previously described [8]. Primary antibodies (all rabbit) were anti-AQP5 (Alomone Labs AQP-005), anti-CAV1 (Abcam ab2910), anti-pro-SFTPC (Millipore AB3786), anti-ACTB (Abcam AB8226), anti-PDPN (Developmental Studies Hybridoma Bank #8.1.1), and anti-LAMIN A/C (sc-20,681, Santa Cruz Biotechnology). Blots were analyzed by chemiluminescence and visualized by West Fempto Super Sensitivity Kit (Thermo Scientific) with a FluorChem 8900 Imaging System (Alpha Innotech).

### Bioelectric properties

Transepithelial electrical resistance (RT, kVcm2) and potential difference (PD, mV) were measured using a Millicell-ERS device (Millipore, Bedford, MA) on Day 6 (D6) of the culture. All RT and PD values were corrected for background levels across blank filters. Equivalent active ion transport rate (i.e., IEQ, mA/cm2) was estimated as PD/RT.

### Immunofluorescence

Freshly isolated hAT2 cells were fixed with 4% paraformaldehyde for 10 min at room temperature (RT), permeabilized with 0.3% Triton, and blocked with CAS blocking reagent (Invitrogen Cat #00–8020, Camarillo, CA) for 30 min at RT. Slides were incubated with mouse anti-VIM (Sigma, #V2258), rabbit-anti-CD45 (Santa Cruz Biotechnology, #sc-25,590), or mouse anti-TTF1(also known as NKX2–1, Novocastra, #NCL-TTF1) antibodies diluted in CAS-block at 4 °C overnight. Slides were then washed in Tris-buffered saline & Tween 20 (TBST) and incubated with goat biotinylated anti-mouse IgM (Vector, #BA-2020), goat biotinylated anti-mouse IgG (Vector, #BA-2000) or goat biotinylated anti-rabbit IgG (Vector, #BA-1000) in CAS-block for 1 h at RT followed by fluorescein avidin D (Vector, #BA-2001). Slides were viewed with a NIKON Eclipse microscope equipped with a QImaging Retica 200R charge-coupled-device camera (QImaging, Surrey, BC, Canada). Florescence intensity was observed and images were processed with Nikon’s software platform, the NIS-Elements Basic Research. Images were captured at 1600X1200 pixels, RGB. The images were then inserted in PowerPoint to generate a Figure. The Figure then was saved as TIFF file and opened in Adobe Photoshop and converted into PDF with resolution > 300 dpi.

### Extraction and processing of RNA for bulk RNA-seq and DNA for whole-genome bisulfite sequencing

1 μg of total RNA was isolated from the indicated AEC using the Illustra TriplePrep Kit (GE LifeSciences, Piscataway, NJ). RNA underwent library preparation and sequencing on the IlluminaHiSeq2000 at the USC Epigenome Core. Briefly, total cell RNA was DNase I digested and then subjected to ribosomal RNA depletion with the Ribominus™ Eukaryote v2 kit (Life Technologies, # A15020, Grand Island, NY). Libraries were constructed with the TruSeq RNA Sample Prep Kit v2 (Illumina # RS-122-2001) and underwent Illumina HiSeq 2000 paired-end sequencing (2 × 50 bp) according to the manufacturer’s instructions as previously reported [59, 112]. Resultant 50 bp paired end FASTQ files were trimmed to remove adapters and realigned to the hg19 genome using Bowtie 2 [113]. Mapped reads were then assembled into transcripts using TopHat v2.0.12 [114]. Resultant reads per kilobase of gene per millions mapped (RPKMs) were used for downstream analysis. Statistical analysis of differential gene expression and correction for covariates including patient sex was performed in DESeq2 [115], and genes located on either the X of Y chromosome were removed due to sex-specific effects on those genes. For whole genome bisulfite sequencing (WGBS), DNA was isolated and library preparation was performed at the USC Epigenome Core. In brief, libraries were plated using the Illumina cBot and run on the Hi-Seq 2000 according to manufacturer’s instructions using HSCS v 1.5.15.1. Bisulfite-treated DNA underwent Paired End 100 cycling. Image analysis and base calling were carried out using RTA 1.13.48.0. Deconvolution and fastq file generation was carried out using CASAVA_v1.7.1a5. Alignment to the genome was carried out using bsmap V 2.5 [116]​. Aligned .bam files were visualized using IGViewer V2.3.40 (Broad Institute, Cambridge MA). Reads were then aligned to the hg19 bisulfite genome and CpG methylation levels and SNPs were determined genome-wide using BisSNP [117]. Methylation domains for each time point during differentiation were calculated using MethylSeekR [16].

### Single cell analysis of published datasets

Single cell datasets were downloaded from publicly available sources, including the IPF Cell Atlas [118], The Molecular Atlas of Human lung [119], and mouse scRNA-seq from the Schiller laboratory [26]. For IPF Cell Atlas, CellRanger matrices were downloaded from GEO (GSE136831). Data were processed using Seurat v 4.1 and the epithelial population subset from immune and fibrotic markers as previously defined [118]. AT1 and AT2 cell clusters were determined using the UMAP projection of normalized and scaled data for expression of known AT2 (*SFTPC*, *SFTPA1*), and AT1 (*AQP5*, *AGER*, *GPRC5A*, *HOPX*), markers. Differential expression between the AT2 and AT1 cell clusters was performed in Seurat using findmarkers. For the Molecular Atlas of human lung and mouse scRNA-seq datasets, supplementary tables where the original publication had defined the genes that represented each cell type were used to subset the 2D AEC RNA-seq data. In the case of the mouse scRNA-seq dataset, only the control (non-bleomycin treated) tables were considered.

### Generation of ChIP-seq and FAIRE from primary human AEC

Chromatin immunoprecipitation (ChIP) was performed using antibodies (Abs) against H3K27Ac (Cat # 39133, Active Motif, Carlsbad CA), H3K4me1 (pAb-037-050) and H3K79me2 (pAb-051-050) from Diagenode (Denville NJ), CTCF (Cat #2899, Cell Signaling, Danvers MA), H3K27me3 (#07–449) and H3K9/14Ac (#06–599) from Millipore (Burlington, MA) and the Imprint Ultra Chromatin Immunoprecipitation Kit (Sigma-Aldrich, St Louis MO). Enrichment for active histone marks in AT1-like cells was verified at the previously identified AT1 cell-type enriched gene *GRAMD2* in a known enhancer region prior to Next-generation sequencing (NGS) library construction. Human *GRAMD2* enhancer primer sequence: Forward 5′-GGTCTCCTGATTTCCTGATG − 3′, Reverse 5′-AGGCTGACTTCTCACTATTC-3′. Enrichment for active enhancer marks in all AEC and for H3K9Ac was also performed prior to NGS library construction at the ubiquitously expressed human *PDGH* gene promoter: Forward 5′- GGTAGGCTACCAGCGGCTCT-3′, Reverse 5′- ACGGTCACGAGAGGAACAGAGGCT-3′. Enrichment of H3K79me2 was performed on Exon 1 of NKX2–1, which was observed previously to be expressed in AT2 and AT1-like cells [8]: Forward 5′-CAAAGAGGACTCCGCTGCTTGTA-3′, Reverse 5′-AGTGACAAGTGGGTTATGTT-3′. Enrichment of CTCF was performed at the CTCF binding site in the intron of *DZIP1L* which has demonstrated CTCF binding in a large number of ENCODE datasets: Forward 5′-TGTTCTGCTGGCCAGATTCG-3′, Reverse 5′-AATGACAACACGACCCTGGAG-3′. Enrichment for H3K27me3 was performed at the *MUC4* locus which we previously observed to be coated with H3K27me3 in AEC [8], Forward 5′-AAACTAGGGACTCCTACTTG-3′, Reverse 5′-GGACAGAATGGGGTGAAT-3′. FAIRE libraries were generated from the histone-depleted supernatants. Free DNA was isolated from the aqueous phase of the phenol-chloroform extraction step [15]. Samples underwent library preparation and 50 bp single end (SE) NGS sequencing using an Illumina HiSeq2000 (Illumina, San Diego CA) at the USC Epigenome Center (USC, Los Angeles CA).

### Peak calling, clustering, and network analysis

Peak calling for histone marks was performed using SICER [120] set to a gap and peak width of 200 bp, except for the H3K27me3 broad mark which had a gap width of 600 bp. Transcription Factor Binding Site (TFBS) analysis was performed with HOMER [121]. Clustering of epigenetic domains was performed using the ‘Diffbind’ package in R (v.1.2.5033) [122]. Specifically, dba.overlap was used to generate a correlational matrix of peak positions, and subsequently dba.plotHeatmap was used for visualization. The Genome Graphs tool, part of the suite of tools available from the UCSC genome browser (www.genome.ucsc.edu) was used to calculate R correlation values. Heatmaps were generated using the ‘gplots’, ‘ComplexHeatmap’, ‘heatmap.2’ and ‘heatmap.plus’ packages in R [123]. 3D plotting was done using ‘plotly’ in R [124]. ROADMAP [90] and ENCODE [88] peaks were downloaded from the Roadmap Epigenome and UCSC genome browser websites, respectively. ROADMAP peaks were previously called using MACS v2.0 [125, 126]. Overlapping H3K27Ac and H3K4me1 regions for each cell type were defined as H3K27Ac peaks with > 50% overlap with H3K4me1. Individual cell type enhancers were then merged into one large enhancer dataset for all cell types (i.e., the “enhance-ome”). ROADMAP lung organ data was the only tissue excluded from analysis because AEC are part of the lung. AEC peak calling was performed again using MACS v2.0 for consistency with Roadmap and ENCODE, with a *p*-value cut off for detection of 1e-3. AEC input DNA was used as background with local bias correction of 5 K and 10 K in the cell type data included. Differential occupancy of AEC enhancer peaks was determined using the UCSC table browser [127]. Peak height was calculated using the area under the curve between the background level and maximal enrichment point along the curve. The ‘PIANO’ package [55] was used in R for gene set enrichment analysis correlation by inputting the list of HOMER-annotated nearest neighbor significantly up- or down-regulated expression datasets with hg19 as the reference genome. Network analysis was performed using the ‘tidyverse’ package in R [68] by summarizing the number of connections between Interrogated Motifs and Associated Motifs. Then, a significance cut-off was applied to retain only those interactions between Interrogated (primary) Motifs and Associated (secondary) motifs above a threshold related to overall enrichment intensity for each cell type (*p* < 10^− 50^ for AT2 cells, p < 10^− 100^ for AT1-like cells). Edgelists were then clustered using the ‘network’ package in R [69] and nodes colored to match the motif families with underlying sequence similarity.

## Supplementary Information

**Additional file 1: Fig. S1.** Quality control for alveolar epithelial cell (AEC) differentiation. A) Western blots examining AT2 and AT1 cell markers during differentiation. LAMIN A/C and ACTB are the loading controls. B) Transepithelial resistance as measured in kΩ-cm^2^ over the course of differentiation. Error bars represent technical duplicates for each plating. C) Representative image of the cytospin staining of AT2 cell specific (TTF1, left panels) and contaminating cell markers (CD45, middle panels; Vimentin, right panels) in freshly isolated AT2 cell preparations from the indicated donors. At least 5 fields were randomly selected for counting. Red = Propidium Iodide, Green = indicated antibody. **Fig. S2.** Concordance of 2D AEC differentiation model with single cell RNAseq on primary lung tissue from multiple consortia. A) Single cell RNAseq analysis derived from control patients listed in IPF Cell Atlas (left) [118]. Cells were filtered based on expression of epithelial markers, specifically clusters containing *EPCAM*, then clustered using Seurat in R. UMAP projections are displayed. Colors indicate cluster identity. UMAP projections from IPF Cell Atlas control epithelial cells (right). Blue = cells with high expression of the indicated marker. Grey = cells lacking expression of the indicated marker. B) Differential expression of AT2 and AT1 enriched gene expression in IPF Cell Atlas plotted by -log10 FDR-corrected significance (left), concordance with differentially expressed genes in the 2D AEC differentiation model (middle). Blue = AT1 enriched genes in IPF Cell Atlas, red = AT2 enriched genes in IPF Cell Atlas. AT1 and AT2-enriched genes from the IPF cell atlas were then subset from the 2D AEC differentiation model RNAseq and plotted as a heatmap (right). Blue = little to no expression, red = high expression. C) Same analysis as for (B) was used on the Molecular Cell Atlas of Human Lung [119]. D) Same analysis was used as for (B) on control mice from lung single cell analysis [26]. **Fig. S3.** FAIRE-seq quality assessment. A) IGV image of FAIRE-seq data. FAIRE was performed on AT2 (D0), AT-transitional phenotype (D4), and AT1-like (D6) cells for Donor 1. Region surrounding FOXA2 locus, which is expressed in both AT2 and AT1-like cells, is shown. FAIRE-seq BigWig tracks are displayed with called FAIRE peaks directly below. B) Table of mapping statistics for FAIRE-seq data. C) Peak saturation plot for FAIRE-seq data. Inset = overlap between regions called FAIRE peaks in AT2 (D0), AT1.5 (D4) and AT1-like cells (D6). **Fig. S4.** H3K4me1 ChIP-seq quality assessment. A) Table of H3K4me1 mapping statistics. B) Peak saturation plot. C) Tag distribution of ChIP-seq read densities at FAIRE peak locations common to both AT2 (D0) and AT1-like (D6) cells. D) Diffbind correlation plot between samples. Condition = Timepoint during differentiation. Black = AT2 (D0), red = AT1.5 (D4), blue = AT1-like (D6). E) Overlap in called peak locations between biological replicates. **Fig. S5.** H3K27Ac ChIP-seq quality assessment. A) Table of H3K27Ac mapping statistics. B) Peak saturation plot. C) Tag distribution of ChIP-seq read densities at FAIRE peak locations common to both AT2 (D0) and AT1-like (D6) cells. D) Diffbind correlation plot between samples. Condition = Timepoint during differentiation. Black = AT2 (D0), red = AT1.5 (D4), blue = AT1-like (D6). E) Overlap in called peak locations between biological replicates. **Fig. S6.** Transcription factor binding site enrichment in separate subsets of A549 enhancers. A) Bar plot of top 25 transcription factor binding site predicted motifs within A549 enhancers subset by overlap with either AT1 enhancers (blue), AT2 enhancers (orange), or only present in A549 (grey). AT1 vs. AT2 enhancer motif correlation = 0.975, *p* < 2.2e^− 16^, AT1 vs. cancer-specific enhancer motif correlation = 0.979, *p* < 2.2e^− 16^, AT2 vs. cancer-specific enhancer motif correlation = 0.930, *p* < 2.2e^− 16^). B) Three-dimensional plot of all HOMER knownMotifs predicted transcription factor binding sites within A549 enhancers overlapping AT1 enhancers (X-axis), AT2 enhancers (Z-axis) or only present in A549 (Y-axis). 2 rotations of the same plot are shown. Colors are scaled from 0 (grey) to 700 (red) on the X-variable (A549 enhancers with AT1 enhancer overlap). **Fig. S7.** Diffbind clustering of individual histone marks. H3K27Ac (purple) and H3K4me1 (green) Diffbind clustering from ROADMAP and AEC tissues. Tissue = Roadmap or AEC indicated cell type. Stage = age of donor, subdivided into pre- and post- natal. **Fig. S8.** Association between cell-type specific enhancers and gene expression. A) Heatmap of expression of genes annotated as the nearest neighbor to cell-type specific enhancers. Rows = number of days during AEC differentiation. Rows were supervised. Columns = genes annotated as nearest neighbor to AEC enhancer regions. Purple = high expression levels, green = low expression levels. B) Heatmap of changes in expression for genes annotated to cell-type specific enhancers utilizing SNPs inside the peaks to correlate with gene expression from lung in the GTEX database. Rows = number of days during AEC differentiation. Rows were supervised. Columns = genes from gene-enhancer pairs significantly associated with SNPs in AEC enhancer regions. Purple = high expression levels, green = low expression levels. **Fig. S9.** AT2 and AT1-like cell-specific enhancers associated with changes in nearby gene expression. Integrative Genomics Viewer (IGV) image of the top AT2 cell enhancer-gene pair, the *SFTPA1/SFTPA2* locus (left) and the top AT1-like cell enhancer-gene pair, the *AASS* locus (right). Bigwig files of RNA-seq and ChIP-seq data from AEC cells are shown, along with regions called peaks directly below the bigwig track. Two regions were identified within the locus as AT1-like cell type specific enhancers. Roadmap (76 samples) and encode (6 samples) peaks were condensed into bed files and merged to create one master enhancer track for non-AEC cell types (presence or absence at any given base). **Fig. S10.** Expression of *FOXA1* and *NKX2–1* in human primary cells from IPF Cell Atlas. A) UMAP projections of *FOXA1* (left) and *NKX2–1* (right) expression across the epithelial populations from data generated in the Banovich/Kropski data [70]. Expression levels are indicated as a color gradient from absent (dark blue) to highly expressed (yellow). For comparative purposes, disease/normal tissue origin as well as cell types as characterized by the Banovich/Kropski groups are shown (right). B) Violin plot of expression for *FOXA1* (left) and *NKX2–1* (right) separated into normal control (blue) and ILD samples (red). All data are available for visualization through the IPF Cell Atlas web browser (http://ipfcellatlas.com/) [70].

**Additional file 2: Supplementary Table S1.** AT1 cell enriched markers across consortia. Genes significantly expressed in AT1 cells from the 2D AEC differentiation model (D0 vs D6, yellow), IPF Cell Atlas (blue), Molecular Atlas of Human Lung (purple), and control treatment AT1 scRNAseq from mouse (orange).

**Additional file 3: Supplementary Table S2.** Enhancer-gene pairs linked in GTEx. Enhancer gene pairs are displayed as the enhancer specific for either “AT2” or “AT-like” cells, alongside the coordinates of the enhancer, the identifier (rsID) of the SNP, the gene name, and the significance of the interaction between the SNP and gene in GTEx. 

**Additional file 4: Supplementary Table S3.** GTEX-annotated enhancer-gene pairs showed differential expression in AEC differentiation. 19 enhancer-gene pairs showed significant differential gene expression during AEC differentiation with log fold change (LogFC) greater than 2.

## Data Availability

All newly generated datasets used in this study are deposited in the public GEO database (GSE150527). ENCODE data, including cell lines and normal tissues data as well as ethanol treated FOXA1 ChIP-seq in A549 cells can be publicly accessed from the UCSC genome browser (genome.ucsc.edu/ENCODE). ROADMAP epigenomics consortium data can be publicly accessed from wwww.roadmapepigenomics.org. ChIP-seq of lentiviral-introduced NKX2–1 in A549 cells was previously published [87]. Single cell RNAseq analysis is available from www.ipfCellAtlas.com (GSE136831), from (https://www.synapse.org/#!Synapse:syn21041850), and from (https://theislab.github.io/LungInjuryRegeneration, GSE141259). All analysis software used to generate used in these analyses are publicly available. All materials are commercially available from the vendors listed in the Materials & Methods section.

## Abbreviations

2D: two-dimensional cell culture
3D: three-dimensional cell culture
AEC: Alveolar epithelial cells
ACTB: Beta Actin
AGER: Advanced glycosylation end-product specific receptor
AP1: Activator Protein 1 transcription factor complex
AT1: alveolar epithelial type 1 cell
AT1-like: alveolar epithelial type 1 – like cell differentiated in culture
AT2: alveolar epithelial type 2 cell
AT-transitional: alveolar epithelial transitional cell type
ATF3: activating transcription factor-3
bHLH: basic helix-loop-helix
BP: base pair
CAV1/2: Caveolin 1/2
ChIP: Chromatin immunoprecipitation
ChIP-seq: Chromatin immunoprecipitation followed by next generation sequencing
CEBPD: CCAAT-enhancer binding protein delta
CLDN18: Claudin 18
CLIC3/5: Chloride intracellular channel 3/5
COL4A1/3: Collagen type IV alpha 1/3 chain
COPD: Chronic obstructive pulmonary disease
CTCF: CCCTC binding factor
D0: Day Zero, start point in culture
D4: Day 4 in culture
D6: Day 6 in culture
DZIP1L: DAZ interacting zinc finger protein 1 like
EDTA: Ethylenediaminetetraacetic acid
EGTA: Egtazic acid
DNA: deoxyribonucleic acid
ENCODE: Encyclopedia of DNA Elements
ES: Embryonic stem cells
ETS: Erythroblast transformation specific
FAIRE: Formaldehyde-assisted Isolation of Regulatory Elements
FOS: FBJ muring osteosarcoma viral oncogene homolog
FOX: forkhead box protein
FOXA1: forkhead box protein A1
FOXA2: forkhead box protein A2
GATA: GATA binding protein
GEO: Gene expression omnibus
GLI: Glioma associated oncogene family
GPRC5A: G-protein coupled receptor class C group 5 member A
HBSS: Hank’s balanced salt solution
H3K27Ac: histone 3 lysine 27 acetylation
H3K27me3: histone 3 lysine 27 trimethylation
H3K4me1: histone 3 lysine 4 monomethylation
H3K9Ac: histone 3 lysine 9 acetylation
H3K79me2/3: histone 3 lysine 79 di- and trimethylation
hAT2: human AT2
HG19: human genome assembly 19
HMEC: human mammary epithelial cells
HOMER: Hypergeometric optimization of motif enrichment
HOPX: Homeodomain only protein
HSF: Heat shock factor
IGFBP7: Insulin like growth factor binding protein 7
IPF: Idiopathic pulmonary fibrosis
iPS: Induced pluripotent stem cells
JUN: V-jun avian sarcoma virus 17 oncogene homolog
LMNA: Lamin A/C
LUAD: Lung adenocarcinoma
LMR: low methylation region
MACS v2.0: Model-based analysis of ChIPseq version 2.0
MEF2: myocyte enhancer factor 2
MUC4: mucin 4
NGS: Next generation sequencing
NF1: Nuclear Factor 1
NFKB: nuclear factor kappa B
NKX: NK2 homeobox family
NKX2–1: NK2 homeobox 1
PBS: Phosphate buffered saline
PDPN: podoplanin
PIANO: Platform for integrative analysis of omics data
PIT1: Pituitary specific positive transcription factor 1
POU2F3: POU class 2 homeobox 3
PMR: partially methylated region
RNA: ribonucleic acid
RNA-seq: ribonucleic acid next generation sequencing
RPKM: Reads per kilobase of gene, per millions mapped
RTKN2: Rhotekin 2
SAM: Sequence alignment map
scRNAseq: single cell RNA-seq
SEMA3B/E: Semaphorni 3B/E
SFTPC: surfactant protein C
SMAD: Mothers against DPP homolog family
SNAIL: Snail homolog 1
SOX: SRY box transcription factor family
SPOCK2: Sparc/osteonectin, cwcv and kazal-like domains proteoglycan 2
STAT: signal transducer and activator of transcription
TCF3/12: transcription factor 3/12
TEAD: Tea domain family member
TEER: Transepithelial electrical resistance
TF: Transcription factor
TFBS: Transcription factor binding site
TWIST: Twist basic helix loop helix
UCSC: University of California Santa Cruz
UMR: unmethylated region
WGBS: whole genome bisulfite sequencing
YAP/TAZ: Yes1 associated transcriptional regulator/WW domain containing transcription factor regulator 1
