# Supplementary material for: Comprehensive epigenomic profiling of human alveolar epithelial differentiation identifies key epigenetic states and transcription factor co-regulatory networks for maintenance of distal lung identity
Source: BMC Genomics. 2021 Dec 18;22:906. doi: 10.1186/s12864-021-08152-6 (PMC8684104; doi:10.1186/s12864-021-08152-6)
Supplement: Supplementary file 1 — Additional file 1: Fig. S1. Quality control for alveolar epithelial cell (AEC) differentiation. A) Western blots examining AT2 and AT1 cell markers during differentiation. LAMIN A/C and ACTB are the loading controls. B) Transepithelial resistance as measured in kΩ-cm2 over the course of differentiation. Error bars represent technical duplicates for each plating. C) Representative image of the cytospin staining of AT2 cell specific (TTF1, left panels) and contaminating cell markers (CD45, middle panels; Vimentin, right panels) in freshly isolated AT2 cell preparations from the indicated donors. At least 5 fields were randomly selected for counting. Red = Propidium Iodide, Green = indicated antibody. Fig. S2. Concordance of 2D AEC differentiation model with single cell RNAseq on primary lung tissue from multiple consortia. A) Single cell RNAseq analysis derived from control patients listed in IPF Cell Atlas (left) [118]. Cells were filtered based on expression of epithelial markers, specifically clusters containing EPCAM, then clustered using Seurat in R. UMAP projections are displayed. Colors indicate cluster identity. UMAP projections from IPF Cell Atlas control epithelial cells (right). Blue = cells with high expression of the indicated marker. Grey = cells lacking expression of the indicated marker. B) Differential expression of AT2 and AT1 enriched gene expression in IPF Cell Atlas plotted by -log10 FDR-corrected significance (left), concordance with differentially expressed genes in the 2D AEC differentiation model (middle). Blue = AT1 enriched genes in IPF Cell Atlas, red = AT2 enriched genes in IPF Cell Atlas. AT1 and AT2-enriched genes from the IPF cell atlas were then subset from the 2D AEC differentiation model RNAseq and plotted as a heatmap (right). Blue = little to no expression, red = high expression. C) Same analysis as for (B) was used on the Molecular Cell Atlas of Human Lung [119]. D) Same analysis was used as for (B) on control mice from lung singl [file 12864_2021_8152_MOESM1_ESM.pdf]

Figure S1

A

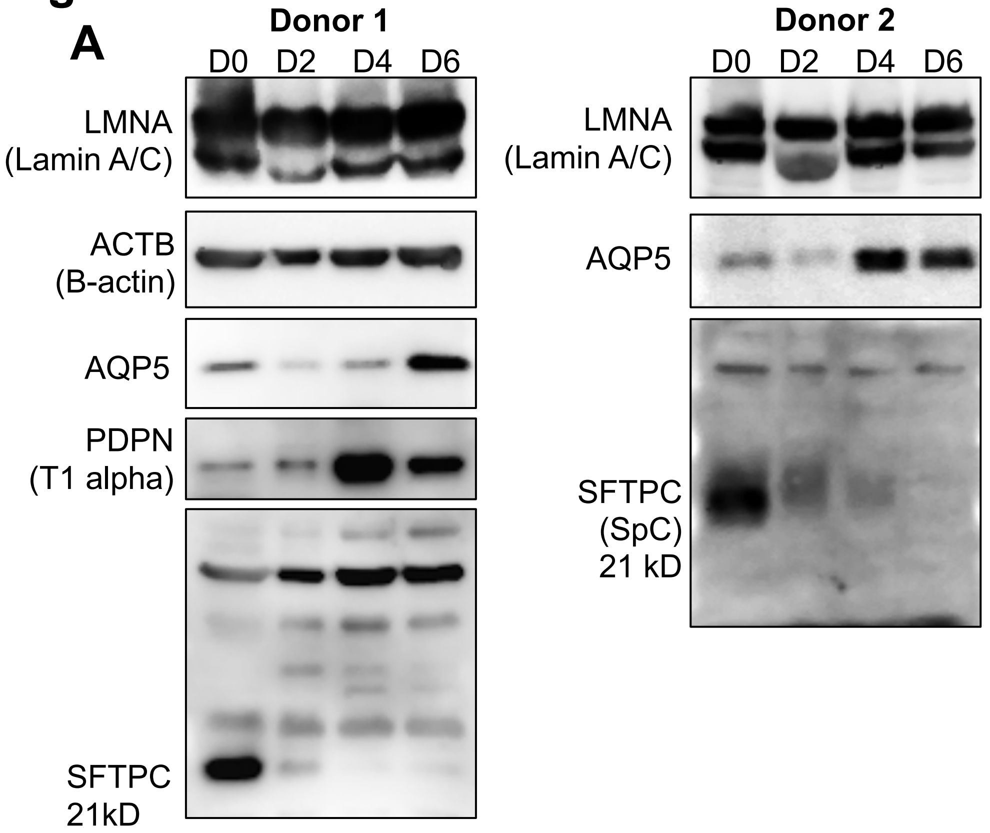

B

Transepithelial Electrical Resistance

Donor 1	D6
RT (KΩ.cm2)	0.40
PD (mV)	3.84
Ieq (μA/cm2)	6.06

Donor 2	D6
RT (KΩ.cm2)	0.52
PD (mV)	4.58
Ieq (μA/cm2)	8.74

C

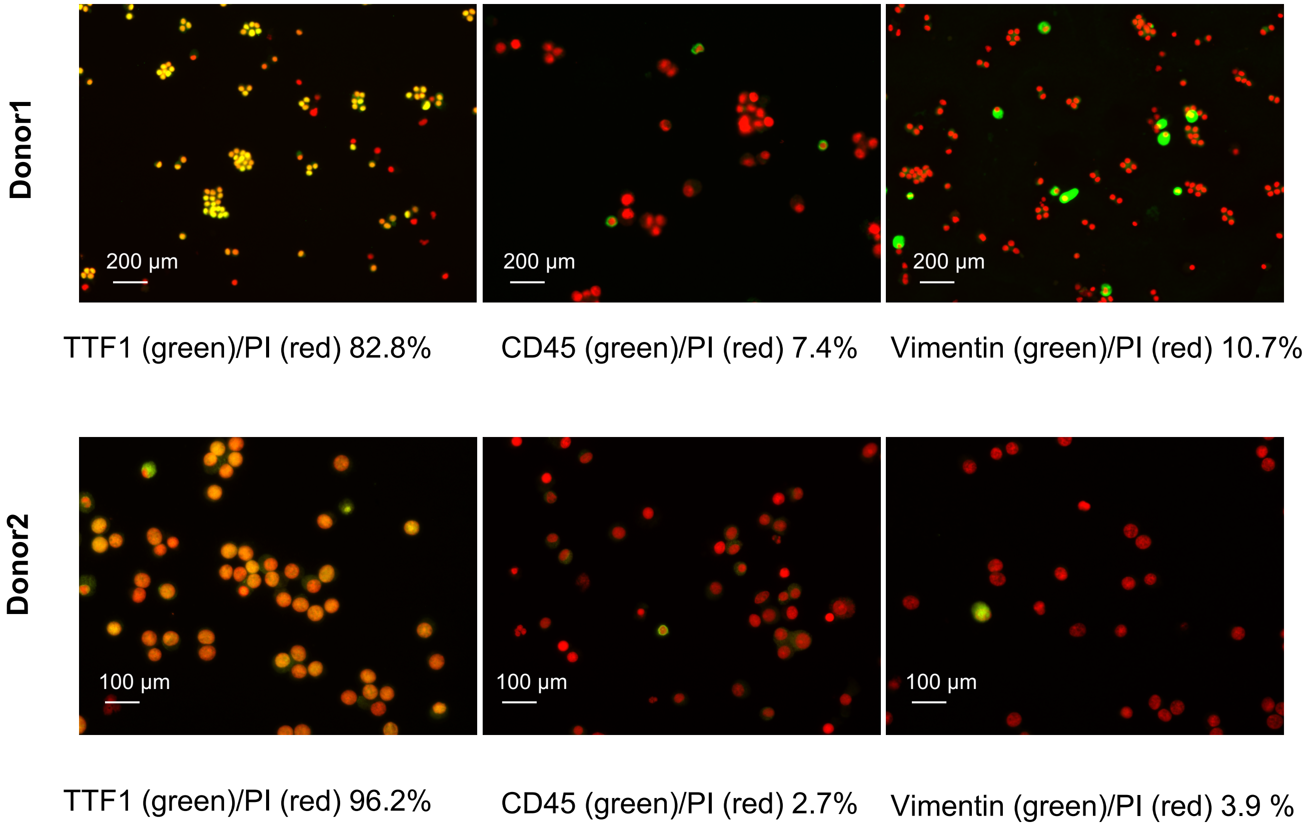

Figure S2

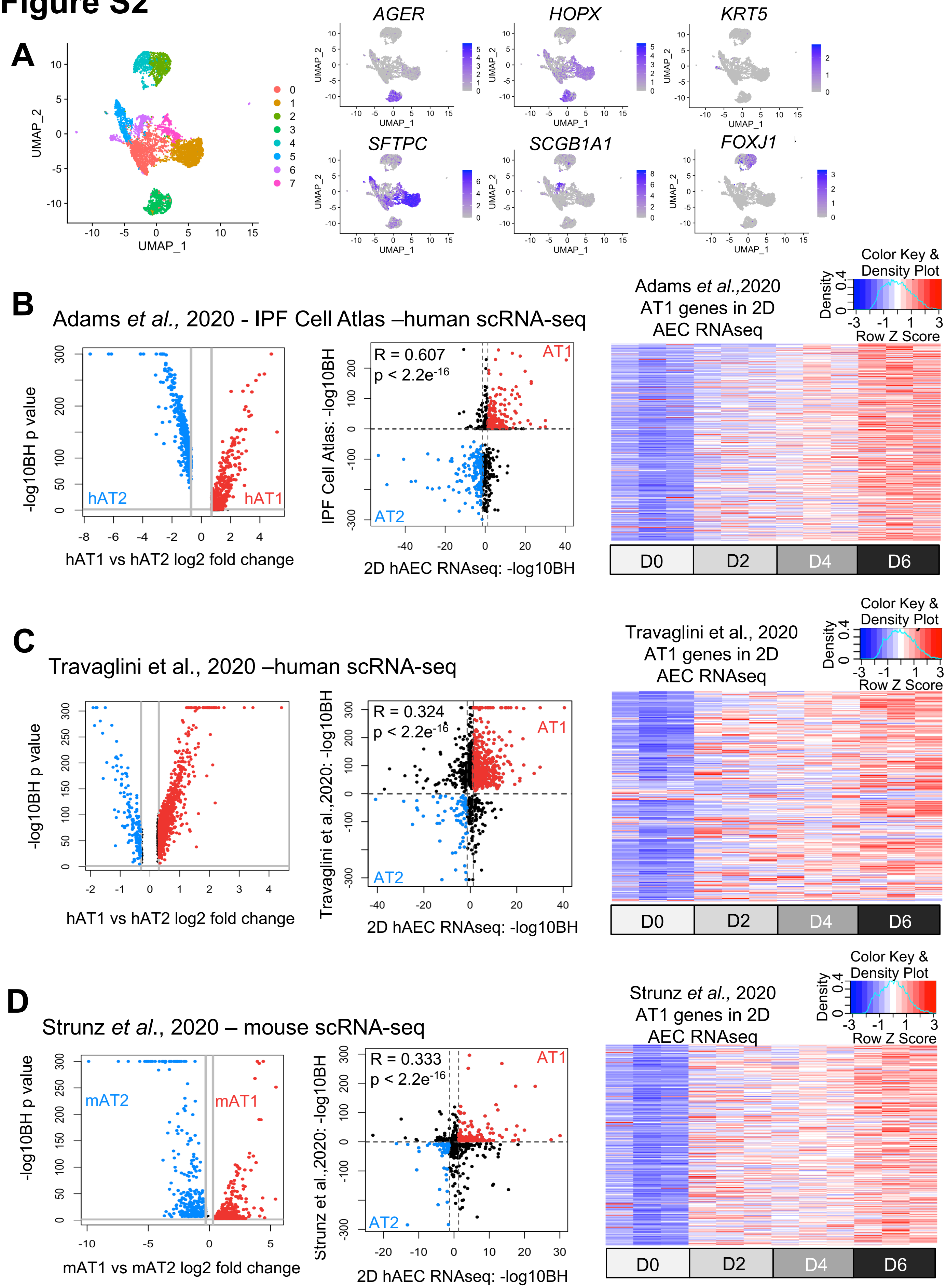
A
UMAP_2
UMAP_1
0
1
2
3
4
5
6
7
AGER
HOPX
KRT5
SFTPC
SCGB1A1
FOXJ1
B
Adams et al., 2020 - IPF Cell Atlas –human scRNA-seq
-log10BH p value
hAT2
hAT1
hAT1 vs hAT2 log2 fold change
R = 0.607
p < 2.2e-16
AT1
AT2
IPF Cell Atlas: -log10BH
2D hAEC RNAseq: -log10BH
Adams et al.,2020
AT1 genes in 2D
AEC RNAseq
Color Key &
Density Plot
Density
Row Z Score
D0
D2
D4
D6
C
Travaglini et al., 2020 –human scRNA-seq
-log10BH p value
hAT1 vs hAT2 log2 fold change
R = 0.324
p < 2.2e-16
AT1
AT2
Travaglini et al.,2020: -log10BH
2D hAEC RNAseq: -log10BH
Travaglini et al., 2020
AT1 genes in 2D
AEC RNAseq
Color Key &
Density Plot
Density
Row Z Score
D0
D2
D4
D6
D
Strunz et al., 2020 – mouse scRNA-seq
-log10BH p value
mAT2
mAT1
mAT1 vs mAT2 log2 fold change
R = 0.333
p < 2.2e-16
AT1
AT2
Strunz et al.,2020: -log10BH
2D hAEC RNAseq: -log10BH
Strunz et al., 2020
AT1 genes in 2D
AEC RNAseq
Color Key &
Density Plot
Density
Row Z Score
D0
D2
D4
D6

Figure S3

A

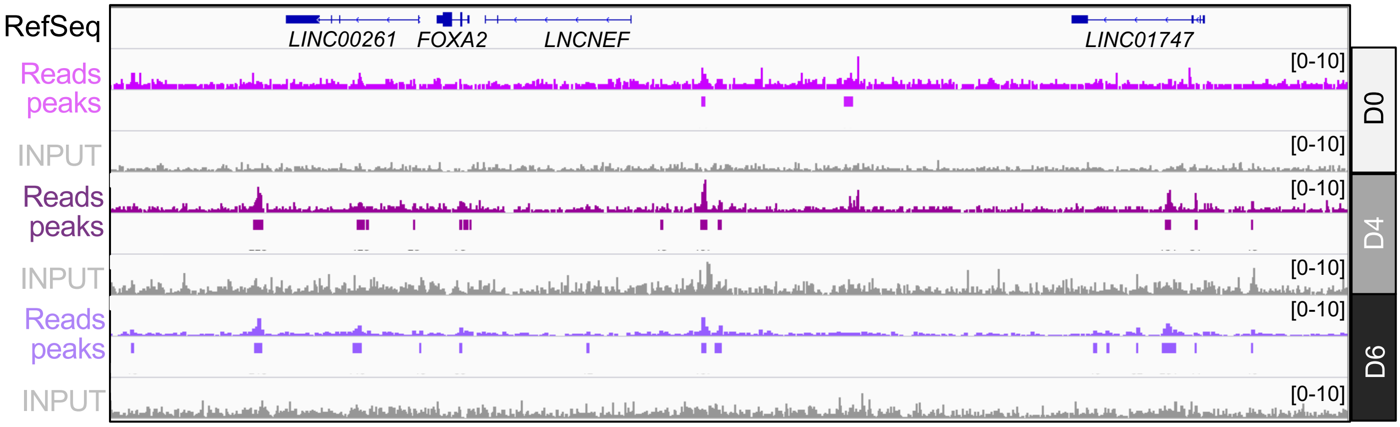

B

	FAIRE		
Day	D0	D4	D6
Lung	Donor 1	Donor 1	Donor 1
# reads	Run1: 51,396,273<br>Run2: 49,753,947	Run1: 53,481,944<br>Run2:52,939,059	Run1: 61,808,878<br>Run2: 60,948,242
# peaks	Run1: 42,753<br>Merged: 57,592	Run1: 59,228<br>Merged: 63,907	Run1: 59,903<br>Merged: 70,901

C

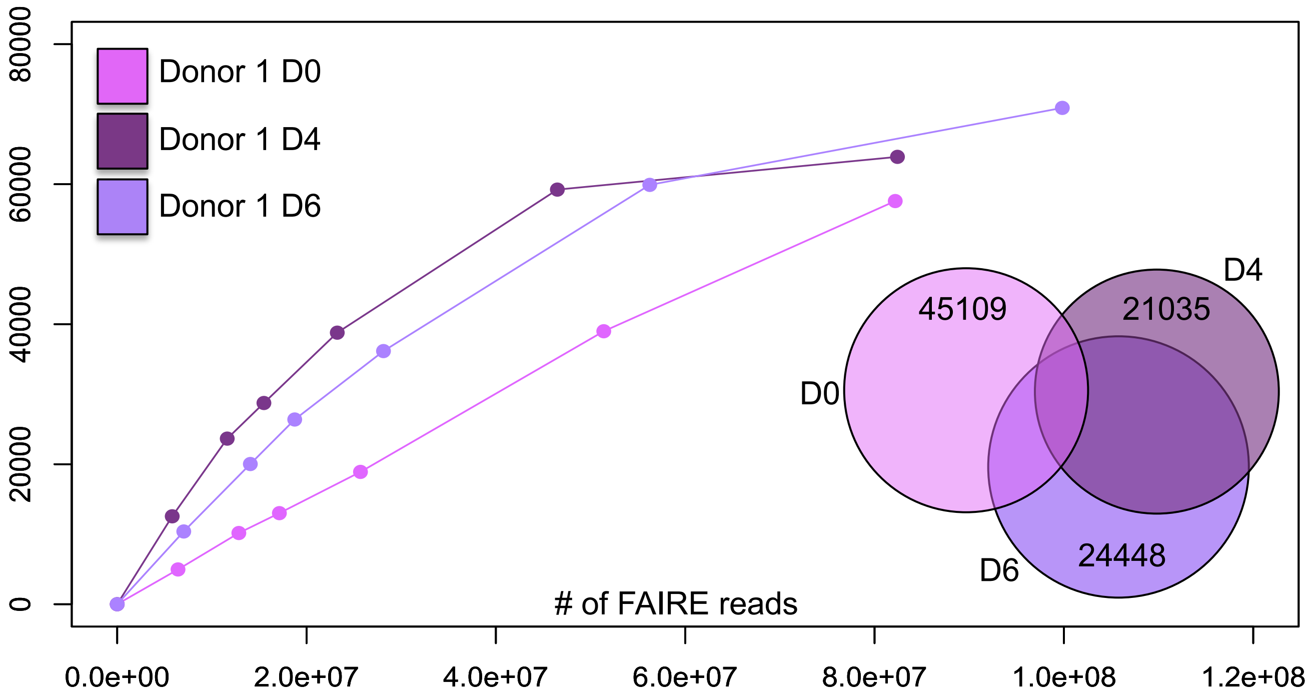

Figure S4

A

	H3K4me1					
Day	D0		D4		D6	
Lung	Donor 1	Donor 2	Donor 1	Donor 2	Donor 1	Donor 2
# reads	R1: 23661212<br>R2: 46284920<br>R3: 31465287	R1:41448100<br>R2:30607744	109244775	R1:31001785<br>R2: 21851830	R1: 22159389<br>R2: 24732185<br>R3: 37091902	R1:32843250<br>R2: 24629851
# peaks	R1: 62755<br>R1+2: 76655<br>All 3: 77353	R1:80529<br>Merged:<br>81979	87202	R1:70932<br>Merged:<br>87000	R1: 52731<br>R1+2: 68618<br>All 3: 89079	R1: 77281<br>Merged: 78574

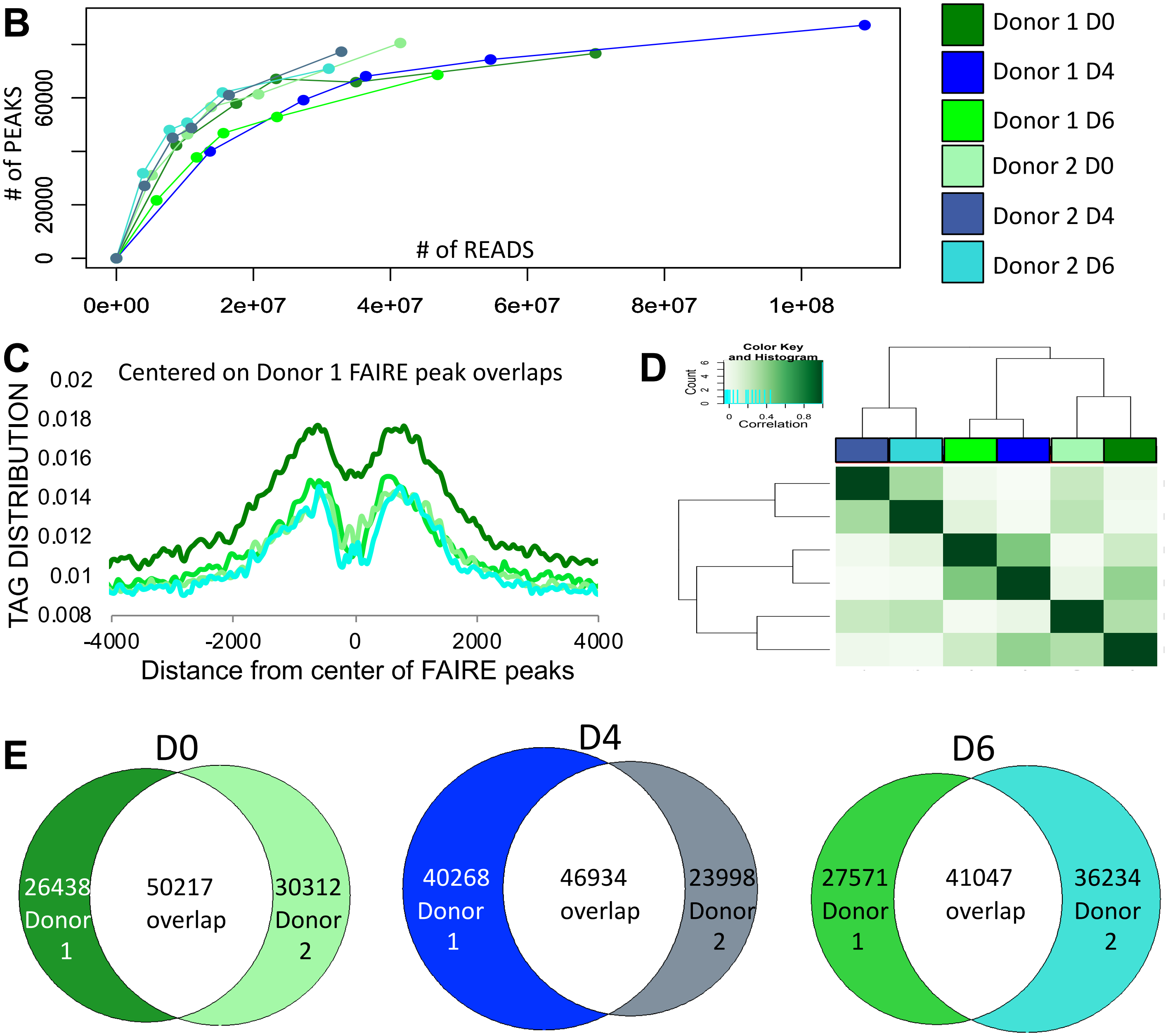

Figure S5

A

	H3K27Ac					
Day	D0		D4		D6	
Lung	Donor 1	Donor 2	Donor 1	Donor 2	Donor 1	Donor 2
# reads	51,381,442	33,486,294	65,963,434	37,340,871	50,241,874	35,428,513
# peaks	39,409	22,241	48,470	36,594	43,144	24,804

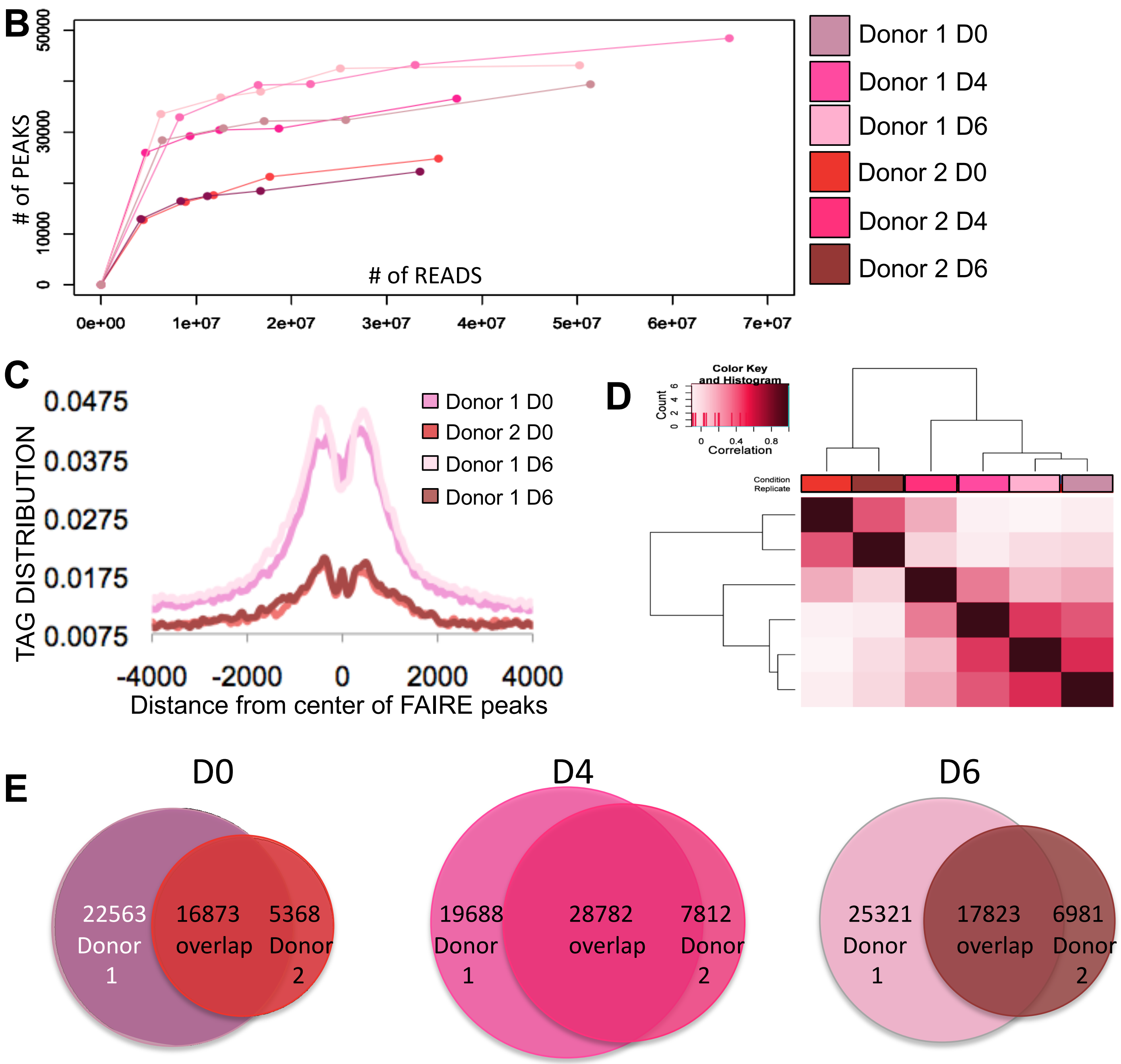

Figure S6

A

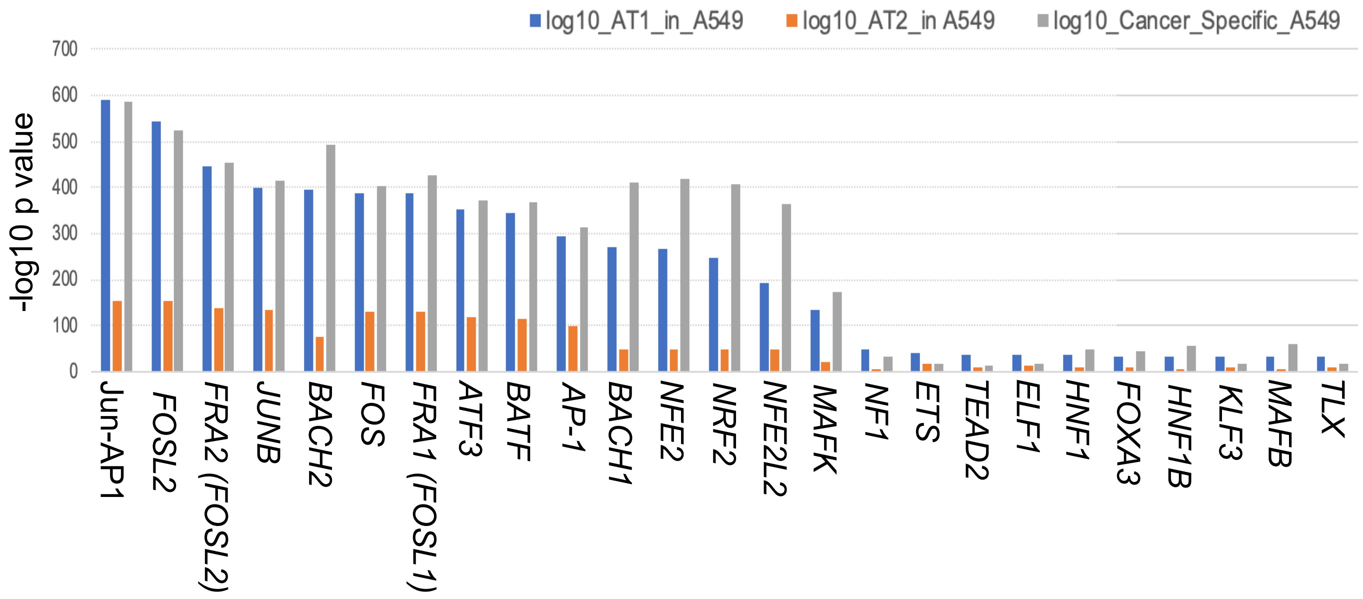

log10_AT1_in_A549
log10_AT2_in A549
log10_Cancer_Specific_A549
-log10 p value
700
600
500
400
300
200
100
0
Jun-AP1
FOSL2
FRA2 (FOSL2)
JUNB
BACH2
FOS
FRA1 (FOSL1)
ATF3
BATF
AP-1
BACH1
NFE2
NRF2
NFE2L2
MAFK
NF1
ETS
TEAD2
ELF1
HNF1
FOXA3
HNF1B
KLF3
MAFB
TLX

B

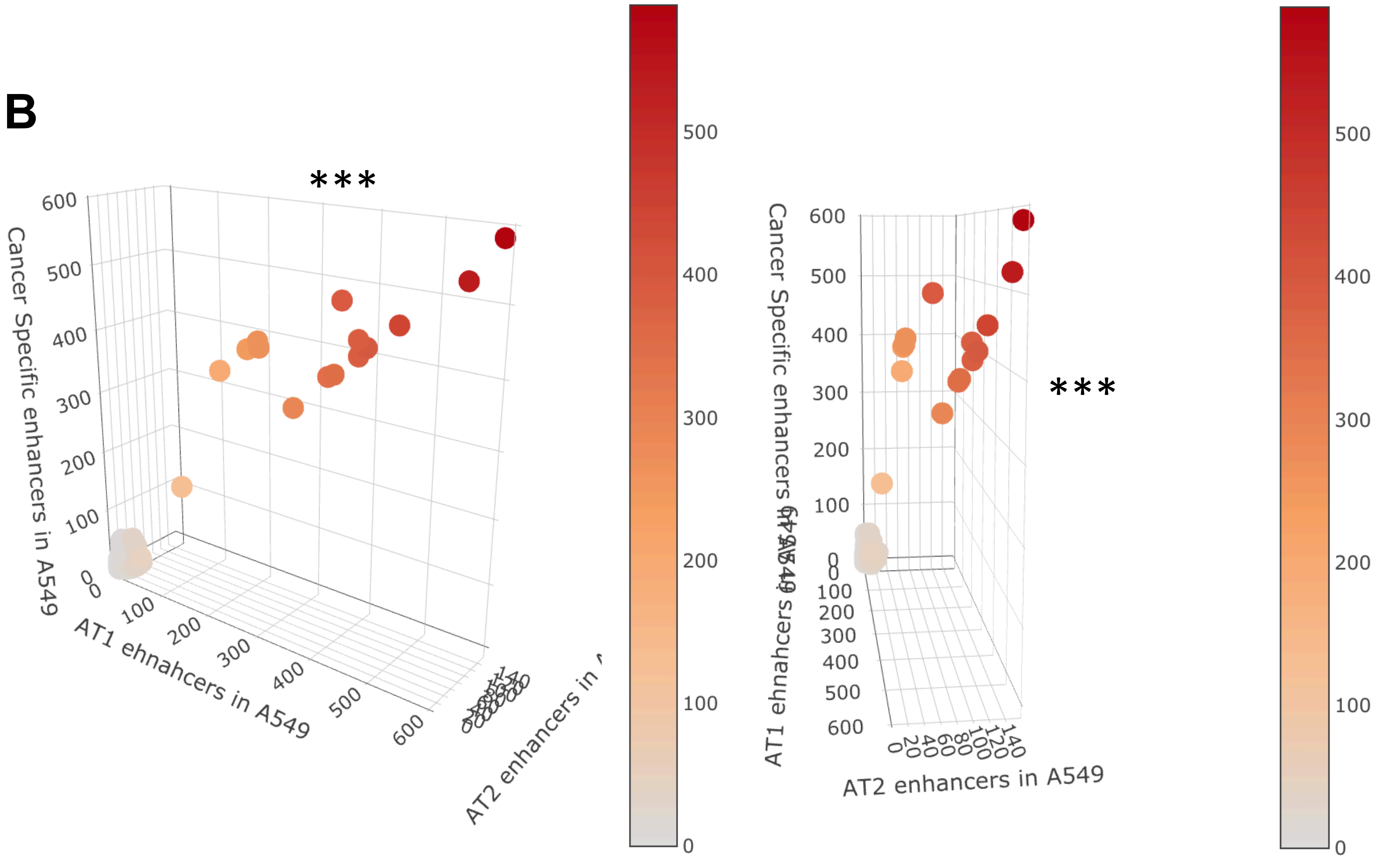

***
Cancer Specific enhancers in A549
AT1 ehnahcers in A549
AT2 enhancers in
Cancer Specific enhancers in A549
AT1 ehnahcers in A549
AT2 enhancers in A549
***
500
400
300
200
100
0

Figure S7

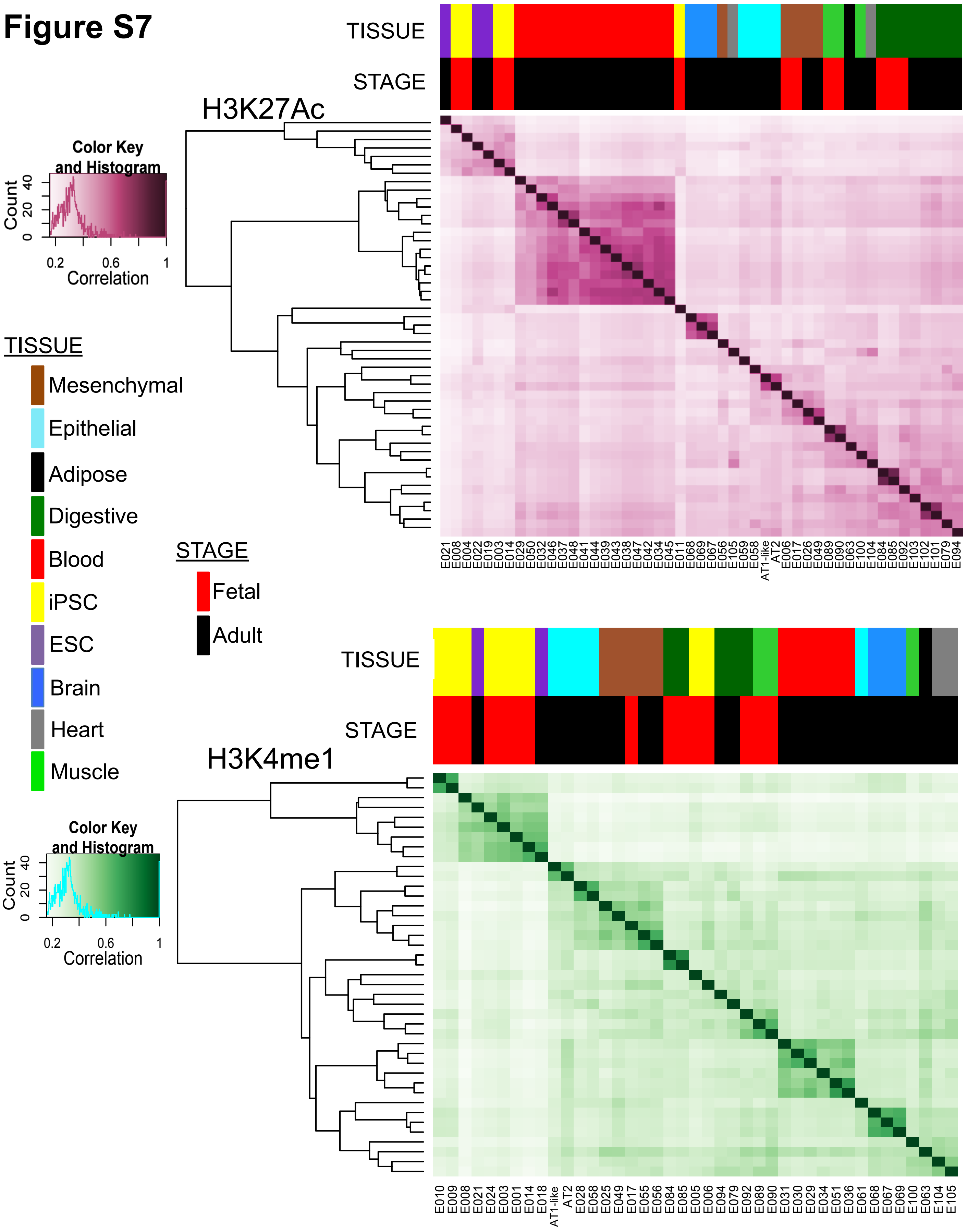

TISSUE
STAGE
H3K27Ac
Color Key and Histogram
Count
0 20 40
0.2 0.6 1
Correlation
TISSUE
Mesenchymal
Epithelial
Adipose
Digestive
Blood
iPSC
ESC
Brain
Heart
Muscle
STAGE
Fetal
Adult
E021 E008 E004 E022 E019 E003 E014 E029 E050 E032 E046 E037 E048 E041 E044 E039 E043 E038 E047 E042 E034 E045 E011 E068 E069 E067 E056 E105 E059 E058 AT1-like AT2 E006 E017 E026 E049 E089 E090 E063 E100 E104 E084 E085 E092 E103 E102 E101 E079 E094
TISSUE
STAGE
H3K4me1
Color Key and Histogram
Count
0 20 40
0.2 0.6 1
Correlation
E010 E009 E008 E021 E024 E003 E001 E014 E018 AT1-like AT2 E028 E058 E025 E049 E017 E055 E056 E084 E085 E005 E006 E094 E079 E092 E089 E090 E031 E030 E029 E034 E051 E036 E061 E068 E067 E069 E100 E063 E104 E105

Figure S8

A

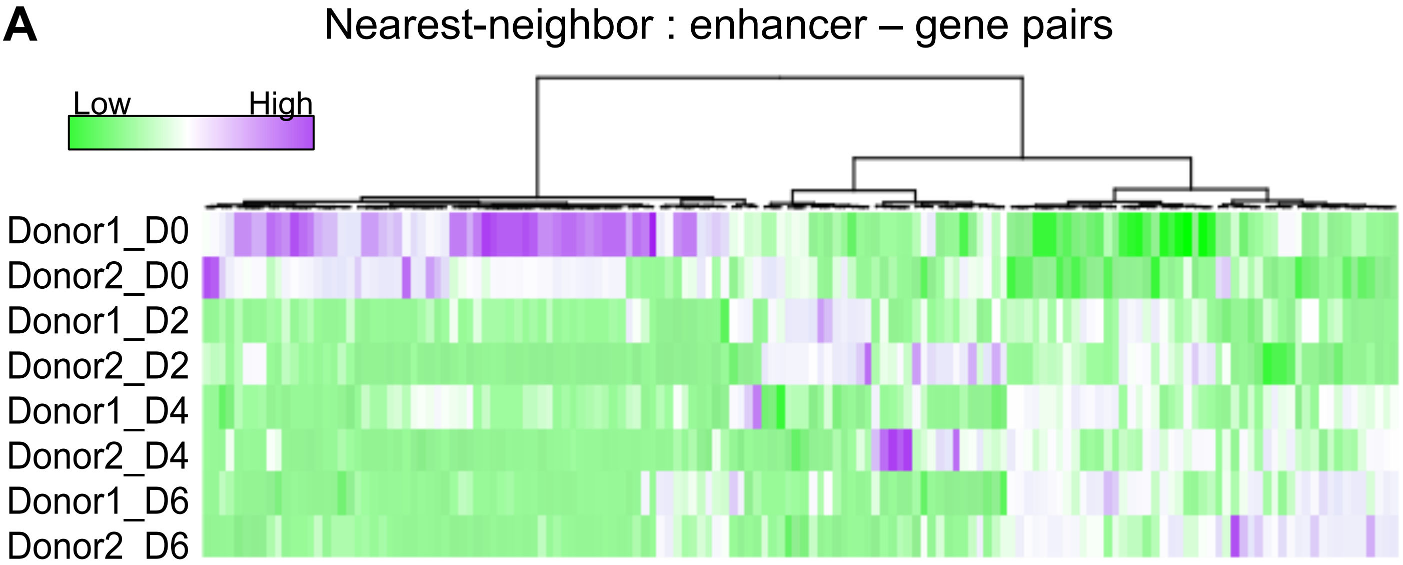
Nearest-neighbor : enhancer – gene pairs
Low
High
Donor1_D0
Donor2_D0
Donor1_D2
Donor2_D2
Donor1_D4
Donor2_D4
Donor1_D6
Donor2_D6

B

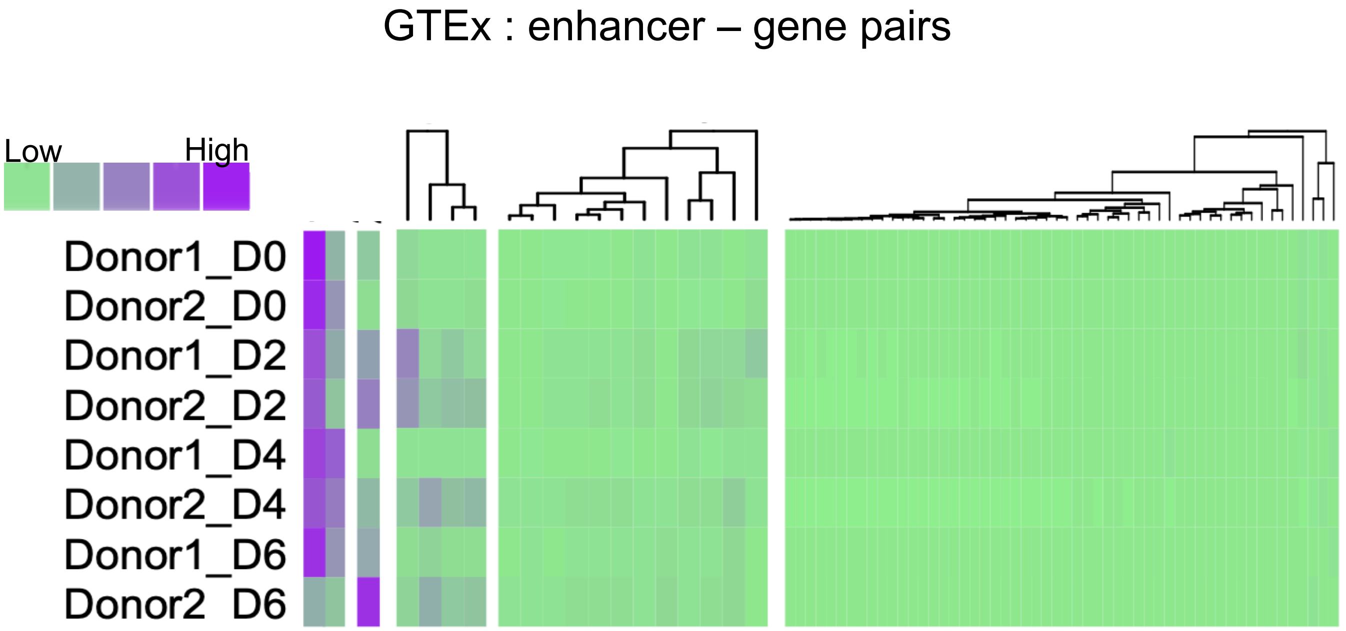
GTEx : enhancer – gene pairs
Low
High
Donor1_D0
Donor2_D0
Donor1_D2
Donor2_D2
Donor1_D4
Donor2_D4
Donor1_D6
Donor2_D6

Figure S9

Top AT2 specific Enhancer-gene pair				
Peak position in hg19			Gene	LogFC
chr10	81345177	81348619	SFTPA2	-3.12

Top AT1 specific Enhancer-gene pair				
Peak position in hg19			Gene	LogFC
chr7	121837600	121841799	AASS	3.06

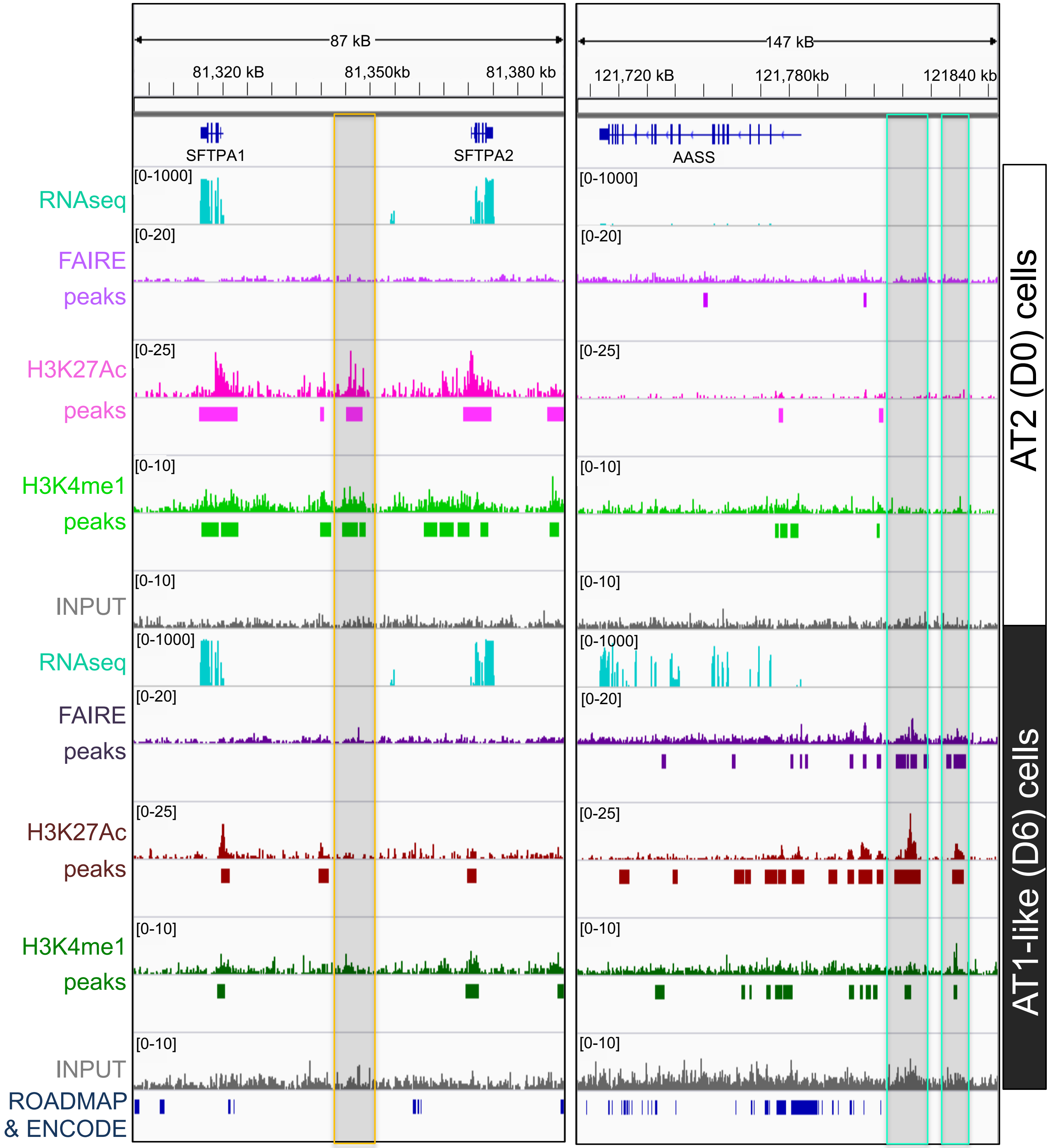

Chr10:81345177-81348619

Chr7:121837600-121841799

Figure S10

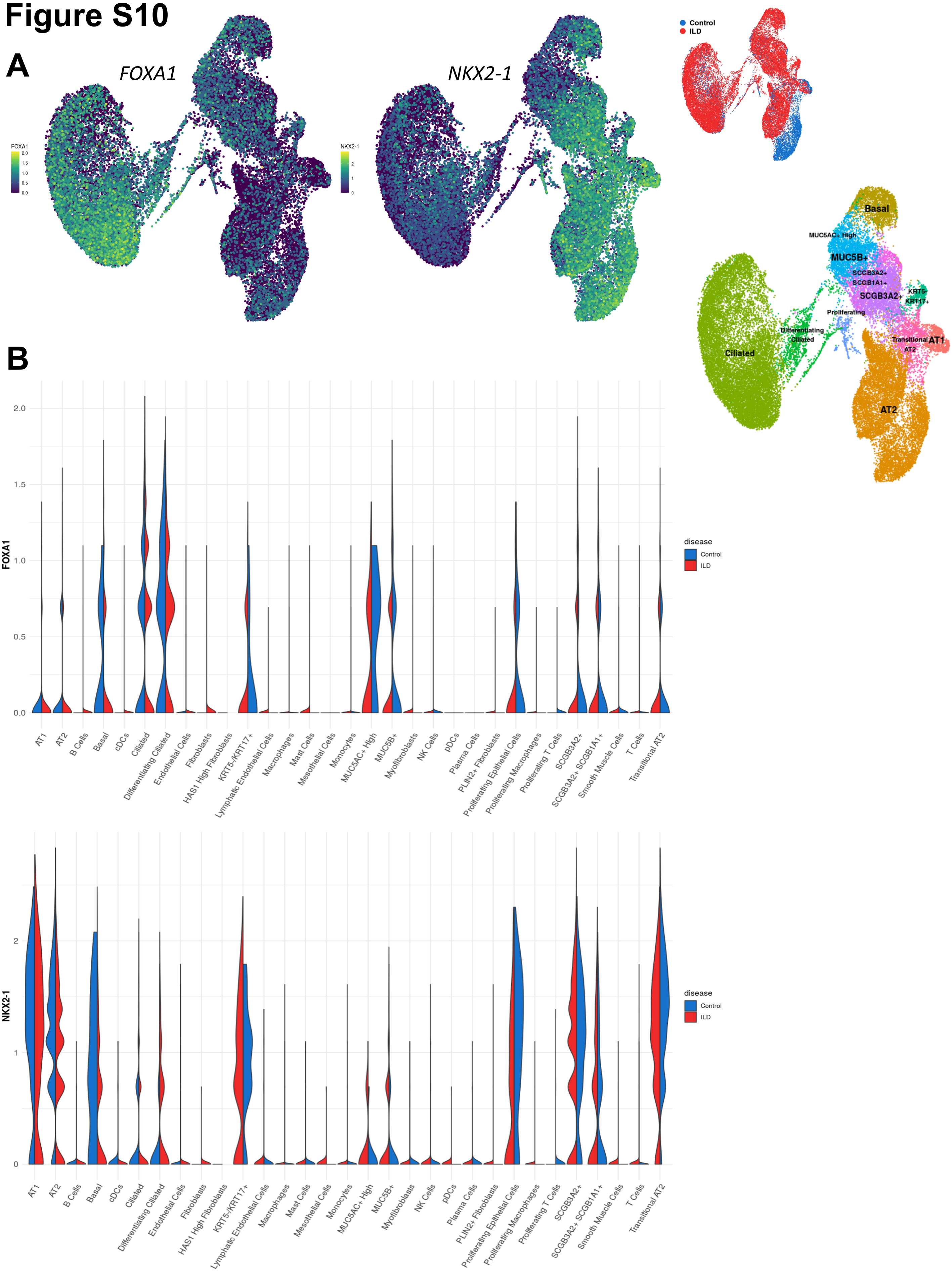
A
FOXA1
NKX2-1
FOXA1
2.0
1.5
1.0
0.5
0.0
NKX2-1
2
1
0
Control
ILD
Basal
MUC5AC+ High
MUC5B+
SCGB3A2+
SCGB1A1+
SCGB3A2+
KRT5-
KRT17+
Proliferating
Differentiating
Ciliated
Ciliated
Transitional
AT2
AT1
AT2
B
FOXA1
disease
Control
ILD
NKX2-1
AT1
AT2
B Cells
Basal
cDCs
Ciliated
Differentiating Ciliated
Endothelial Cells
Fibroblasts
HAS1 High Fibroblasts
KRT5-/KRT17+
Lymphatic Endothelial Cells
Macrophages
Mast Cells
Mesothelial Cells
Monocytes
MUC5AC+ High
MUC5B+
Myofibroblasts
NK Cells
pDCs
Plasma Cells
PLIN2+ Fibroblasts
Proliferating Epithelial Cells
Proliferating Macrophages
Proliferating T Cells
SCGB3A2+
SCGB3A2+ SCGB1A1+
Smooth Muscle Cells
T Cells
Transitional AT2
